# Evolution of acid nociception: ion channels and receptors for detecting acid

**DOI:** 10.1098/rstb.2019.0291

**Published:** 2019-09-23

**Authors:** Luke A. Pattison, Gerard Callejo, Ewan St John Smith

**Affiliations:** Department of Pharmacology, University of Cambridge, Tennis Court Road, Cambridge CB2 1PD, UK

**Keywords:** acid, acid-sensing ion channel, TRP channel, two-pore potassium channel, proton-sensing GPCR, nociception

## Abstract

Nociceptors, i.e. sensory neurons tuned to detect noxious stimuli, are found in numerous phyla of the Animalia kingdom and are often polymodal, responding to a variety of stimuli, e.g. heat, cold, pressure and chemicals, such as acid. Owing to the ability of protons to have a profound effect on ionic homeostasis and damage macromolecular structures, it is no wonder that the ability to detect acid is conserved across many species. To detect changes in pH, nociceptors are equipped with an assortment of different acid sensors, some of which can detect mild changes in pH, such as the acid-sensing ion channels, proton-sensing G protein-coupled receptors and several two-pore potassium channels, whereas others, such as the transient receptor potential vanilloid 1 ion channel, require larger shifts in pH. This review will discuss the evolution of acid sensation and the different mechanisms by which nociceptors can detect acid.

This article is part of the Theo Murphy meeting issue ‘Evolution of mechanisms and behaviour important for pain’.

## 1. Nociception evolution and the drive for nociceptor acid-sensitivity

In the 160th anniversary of their publication, Charles Darwin's words still ring true, ‘any variation…if it be in any degree profitable to an individual of any species…will tend to the preservation of that individual, and will generally be inherited by its offspring’ [1, p. 61]. It could be argued that one of the most profitable facets of any organism is the ability to detect and react to potentially damaging stimuli in its environment, hence nociception (derived from the Latin *nocere* meaning to hurt/harm), the neural process of encoding noxious stimuli, is common to many species in the Animalia kingdom [2–6]. However, not all Animalia have a complex nervous system, for example, Porifera (sponges) contract in response to changes in extrinsic conditions (e.g. turbulent water) and glass sponges transmit electrical signals through their syncytial tissues [7]. Although a number of genes associated with neuronal function have been identified in the sponge *Amphimedon queenslandica* [8], as is the case with Placozoa [9], the presence of neuronal genes and electrical conductivity does not constitute a nervous system and experimental work to determine if neuronal gene expression is linked to sensory function in Porifera and/or Placozoa remains to be determined [10]. By contrast, Cnidaria (e.g. jellyfish and sea anemones), possess diffuse nerve nets [11] and mechanical stimulation of *Calliactis parasitica* produces nervous impulses, strong stimulation (i.e. potentially nociceptive) evoking a closure reflex [12]. Similarly, Ctenophores (comb jellies) also possess sensory receptors and nerve cells [13], but there has, to our knowledge, been no investigation of the potential nociceptive function of their nervous system. It is therefore in Bilateria (e.g. Animalia other than Porifera, Placozoa, Ctenophores and Cnidaria) where an integrated nervous system has fully evolved [14] and nociception has been most frequently studied. In humans, the importance of a nociceptive system is illustrated by individuals with congenital insensitivity to pain, who often accumulate injuries and whose heightened risk-taking behaviour is thought to contribute to higher early-life mortality [15]. There are also genetic variations that result in excessive nociception and studying these variations at a functional level has contributed to understanding of how the nociceptive system works, as well as highlighting points for therapeutic intervention [16]. It should be noted that nociception and pain are not the same, even though the terms are often used interchangeably. As above, nociception is the neural process of encoding noxious stimuli, which involves specialized sensory neurons called nociceptors. By contrast, pain is usually defined as an unpleasant sensory and emotional experience associated with actual or potential tissue damage, or described in terms of such damage. Using the term pain (rather than nociception) for non-mammalian species has produced rigorous discussion in the field owing to the debate over which organisms have the capacity for emotional processing, however, this is beyond the scope of this article and has been reviewed elsewhere [17–19].

In many species, nociceptors are polymodal, i.e. they respond to multiple stimuli (e.g. heat, pressure and chemicals such as acid), owing to the expression of different receptors. Polymodality has been determined using a range of electrophysiological and imaging approaches, and recent single-cell RNA-sequencing studies show that sensory neurons usually express a multitude of different receptors that confer polymodality and enable transcriptomic segregation of sensory neurons into subtypes, whose function can be interrogated *in vitro* and *in vivo* [20–24].

**Figure 1.**
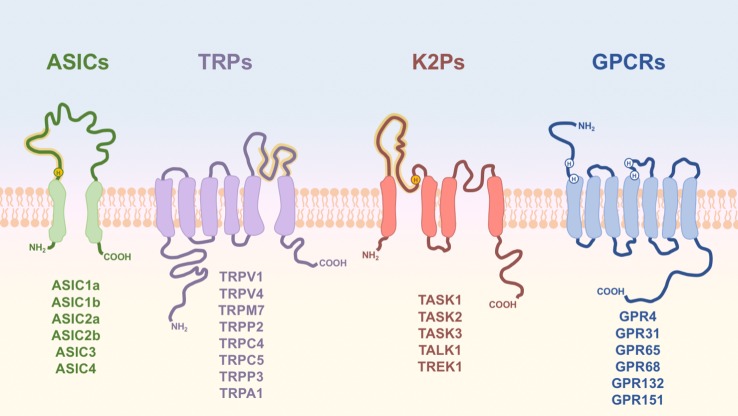
Membrane topologies of proton-sensitive receptor subunits. Schematic diagram of the basic structure of proton-sensitive receptors, with residues or regions important for proton sensitivity annotated (yellow—highly conserved among family members; white—less conserved or important in some, but not all, family members). Functional ASICs, K2Ps and TRPs are multimeric, but for simplicity only one subunit of each receptor is shown.

**Figure 2.**
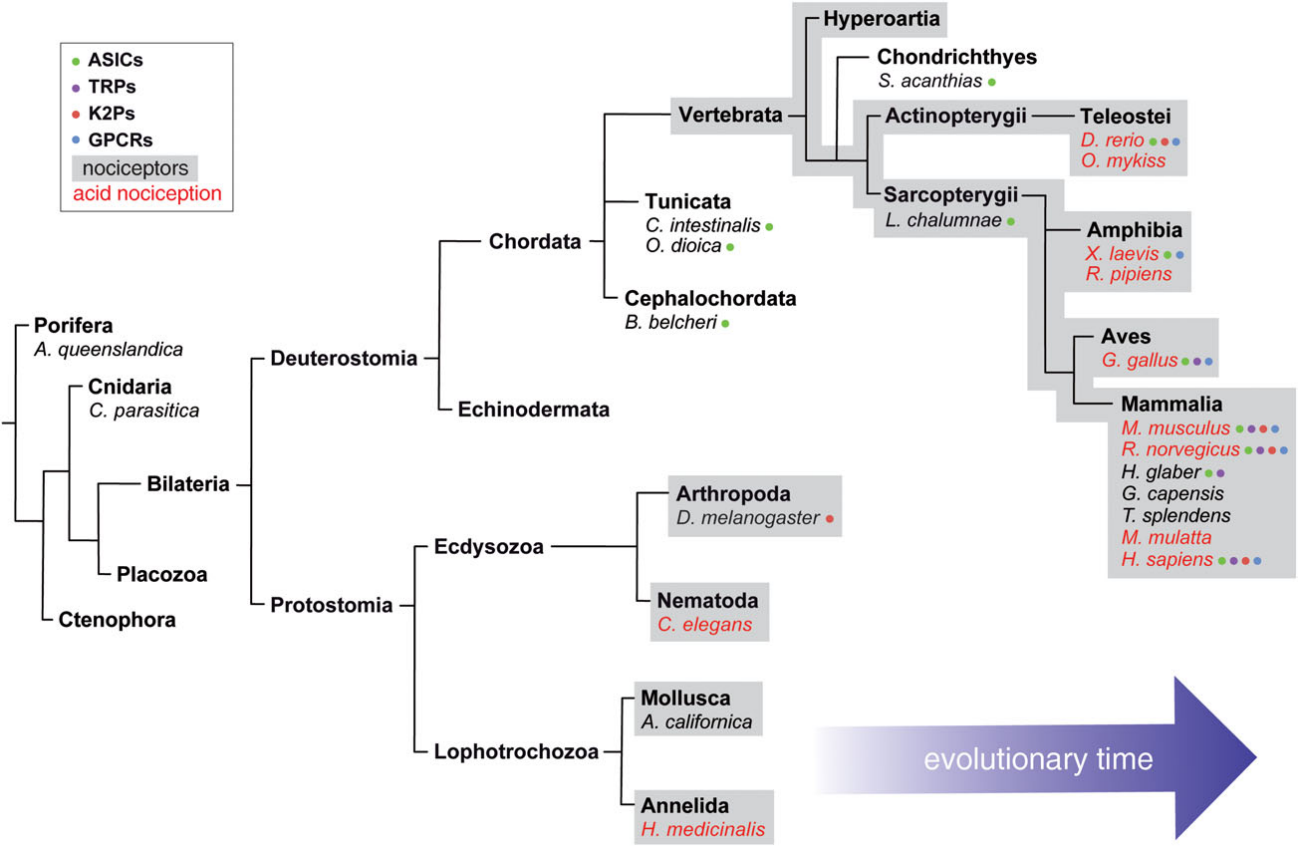
Phylogeny of general nociception and acid nociception. Annotated phylogenetic tree indicating the presence of general nociceptors, observation of acid nociception and functional expression of proton-sensitive receptors. Annotation is limited by the rarity of molecular studies focusing on lower-order species. Expression of proton-sensitive receptors is only acknowledged for those species where proton sensitivity of at least one member of the group in question has been empirically proven. For simplicity only species addressed in this review are shown.

Here, we will focus on proton-induced nociceptor activation, others having previously reviewed sensory neuron mechanosensitivity [25–27] and thermosensitivity [28,29]. Protons influence ion homeostasis and modulate enzyme activity, and thus organisms have evolved the ability to regulate extracellular and intracellular pH through membrane transporters and a range of proton buffering systems [30–32]. Expression of a range of proton-sensitive receptors, summarized in figure 1, permits detection of protons by nociceptors, proton-induced activation/inhibition of these receptors can in turn modulate nociceptor excitability. The ability of protons to activate nociceptors and/or evoke nocifensive behaviour has been demonstrated in a wide range of species, including: the nematode worm *Caenorhabditis elegans* [33], the medicinal leech *Hirudo medicinalis* [34], the northern grass frog *Rana pipiens* [35–37], the rainbow trout *Oncorhynchus mykiss* [38,39], the chicken *Gallus gallus* [40,41], the mouse *Mus musculus* [42,43], the rat *Rattus norvegicus* [44] and the human *Homo sapiens* [45,46]; however, acid nociception is not universal, the naked mole-rat (*Heterocephalus glaber*), the Cape mole-rat (*Georychus capensis*) and the East African root rat (*Tachyoryctes splendens*) displaying no acid-induced nocifensive behaviour [42,47]. The presence of acid nociception in such a wide variety of species, both aquatic and terrestrial, demonstrates the likely evolutionary pressure to maintain selection for being able to detect and respond to changes in the pH of an organism's environment, whereas presumably any cost to those organisms that do not display acid nociception is outweighed by some other benefit. A phylogenetic summary depicting the evolution of nociceptors, acid nociception and different acid-sensors is illustrated in figure 2.

In humans, perhaps the first demonstration that acid evokes pain was from von Gaza and colleagues who reported that pain and a change in the proton concentration were common to inflammation, and that tissue alkalinization could reverse pain associated with abscesses [48]. Indeed, work in humans [49–52] and rodents [53] supports the fact that tissue acidosis occurs during inflammation, but equally inflammation can occur in the absence of acidosis [54,55]. In terms of the mechanisms by which acid causes pain, Krishtal and colleagues were the first to demonstrate that protons could excite sensory neurons by evoking transient inward currents [56]. Subsequent analysis of various mammalian nociceptors demonstrated that protons produce three main types of excitation: transient inward currents (current inactivation in the presence of protons), sustained inward currents (continuous inward current in the presence of protons) and mixed (both transient and sustained phases) [46,57–61]. Underpinning these different responses are a variety of different mechanisms and this review will discuss the different mechanisms and what is understood about their roles in different species.

## 2. Acid-sensing ion channels

The acid-sensing ion channels (ASICs) are part of the epithelial sodium channel (ENaC)/degenerin (DEG) ion channel superfamily, which in mammals consists of nine genes, four encode ENaC subunits, four encode ASICs and one encodes the bile acid-sensitive ion channel (BASIC, sometimes termed ASIC5, approx. 30% homologous to other ASICs, but proton-insensitive [62]). The four ASIC genes encode six ASIC subunits, splice variants of the ASIC1 and ASIC2 genes resulting in: ASIC1a [63], ASIC1b [64,65], ASIC2a [66], ASIC2b [67], ASIC3 [68] and ASIC4 [69,70]. Although there was initial debate surrounding the subunit stoichiometry of functional ASICs, both X-ray crystallography [71] and atomic force microscopy [72] have demonstrated that ASICs are trimeric ion channels. However, not all ASIC subunit configurations produce proton-sensitive ion channels, ASIC2b and ASIC4 homomers are proton-insensitive, but can form proton-sensitive heteromers and/or regulate ASIC subunit surface expression [67,73]; the naked mole-rat ASIC3 is also proton-insensitive, a potential adaptation to a subterranean lifestyle [74]. When proton-sensitive ASICs are activated, an inward cation flux (largely Na^+^, although ASIC1a shows Ca^2+^ permeability) leads to neuronal depolarization and in nociceptors, if of sufficient magnitude to produce action potential firing, would lead to nociception. With regard to their expression profile, in mammals, all ASIC subunits are expressed in sensory neuron cell bodies in the dorsal root ganglia (DRG), albeit that ASIC4 is expressed at comparatively much lower levels [22,23,75]; interestingly the ASIC3 transcript is downregulated in sensory neurons of the proton-insensitive rodents, the naked mole-rat, the Cape mole-rat and the East African root rat [47].

The chicken ASIC1a crystal structure identified a region termed the acidic pocket containing three carboxylate pairs (D238–D350, E239–D346 and E220–D408; chicken ASIC1a numbering), which were suggested to be the primary sites for proton sensing [71]. Mutational analysis shows that these residues, while regulating pH sensitivity, do not fully abolish ASIC1a proton sensitivity [76,77]. Moreover, the proton-sensitive ASIC2a lacks D350, which might explain why it is the least proton sensitive of the functional mammalian ASIC homomers [78], but ASIC2b, which is proton-insensitive, also only lacks D350 [79]. Together, these results suggest that sites outside of the acidic pocket are important for ASIC proton sensitivity and several studies have identified further amino acids that are required for normal proton sensing by ASIC1a [76,77,80,81]. Furthermore, comparative analysis of rat ASIC2a/ASIC2b [82] and zebrafish (*Danio rerio*) zASIC4.1 (proton-sensitive) and zASIC4.2 (proton-insensitive) [83] have demonstrated the critical importance of the extracellular domain proximal to the first transmembrane domain for conferring ASIC proton sensitivity, and in particular the importance of the histidine residue H73 (mouse ASIC1a numbering) (figure 1) [79,84]. In first attempting to determine when proton sensitivity arose in ASICs, it was demonstrated that the spiny dogfish (*Squalus acanthias*, a cartilaginous fish) produces proton-sensitive ASICs [85], but that neither the lamprey *Lampetra fluviatilis* [86], nor the tunicate *Ciona intestinalis* [87] do. However, more recent analysis of ENaC/DEG sequences from several phyla has demonstrated that ASICs from a variety of deuterostome lineages are not only expressed in the nervous system, but are also proton-sensitive (including the tunicate *Oikopleura dioica*) [88]. It was also demonstrated that the conserved H73 residue (mouse ASIC1a numbering), proximal to the first transmembrane domain, was critical in determining proton sensitivity in both the lancelet *Branchiostoma belcheri* and mice (i.e. distantly related species), suggesting that the appearance of this histidine coincided with the emergence of ASIC proton sensitivity with further lineage specific changes occurring over time [88]. Further evidence for the importance of the extracellular domain proximal to the first transmembrane domain comes from studying ASIC4, whereby west Indian Ocean coelacanth (*Latimeria chalumnae*), African clawed frog (*Xenopus laevis*) and chicken (*G. gallus*) ASIC4s all respond to protons, but rat ASIC4 does not: 24 amino acids in the β1 strand running from the first transmembrane domain into the extracellular domain (including H73) were shown to confer proton sensitivity and insertion of a single amino acid in mammalian ASIC4 resulted in proton-insensitivity [88]. Overall, the extensive recent analysis by Lynagh *et al*. [88] clearly demonstrates that ASIC proton sensitivity is conserved across many animal phyla, including invertebrates, although little is known about the contribution to nociception of ASICs in these species.

When considering the ENaC/DEG family more broadly, members such as HaFaNaC from the mollusc *Cornu aspersum* (previously *Helix aspersa*) [89] and HyNaC from the cnidarian *Hydra magnipapillata* [90] are activated by peptides, rather than protons, suggesting a potential role for evolutionary ASIC precursors as peptide sensors; indeed, mammalian ASICs are modulated, but not activated, by a variety of peptides [91–95]. Interestingly, the non-proton agonist of ASIC3 2-guanidine-4-methylquinazoline activates HaFaNaC and related mollusc ENaC/DEG channels by a mechanism distinct to their activation by the endogenous agonist FMRFamide [96], which highlights that dual activation/modulation of ASICs and related channels is a conserved feature.

In addition to peptides, ASIC function can be modulated by numerous endogenous mediators and other compounds (see [97,98] for a review), some of which, like arachidonic acid [44,57,99], nitric oxide [100] and protein kinase C (PKC) [101], are, like protons, upregulated in inflammation and thus probably work synergistically to activate ASICs and produce pain. In terms of how protons modulate mammalian nociceptor function, they both activate and sensitize rodent nociceptors [58,102], ASIC3 being particularly important. For example, protons activate mouse C-fibre nociceptors in an ASIC3-dependent manner, mice lacking ASIC3 showing less nociceptor firing at pH 5.0 than wild-type mice (although no difference was observed at pH 4.0 and ASIC3^−/−^ mice showed no difference in acid-evoked licking behaviour) [43]. Similarly in rats, blockade of ASIC3 with APETx2 inhibits acid-evoked nociceptor firing and reduces acid-evoked pain behaviour [44,103], but a caveat of interpreting this is that APETx2 also inhibits the voltage-gated sodium channel subunit 1.8 (Na_V_1.8) [104]. Considering the role of tissue acidosis in some forms of inflammation, there has been considerable investigation of how ASICs contribute to hyperalgesia in numerous animal models and overall evidence supports the targeting of ASICs to relieve pain [105,106]. Although evidence supports targeting of ASIC1a/1b subunits [107,108], there has been more extensive investigation of ASIC3, most likely owing to its activation producing a pronounced sustained phase following the initial transient phase, i.e. ASIC3 can probably transduce sustained tissue acidosis into nociceptor activation and pain behaviour [68]. For example, arachidonic acid potentiates the sustained phase of ASIC3 [57], arachidonic acid potentiates acid-evoked pain in rats that is reversed by ASIC3 inhibition [44], chronic hyperalgesia induced by repeated intramuscular acid injections is abolished in mice lacking ASIC3 [109] and a peptide from *Conus textile* venom potentiates ASIC3 activity concomitant with enhancing acid-evoked hyperalgesia [94].

In humans, there is some evidence to support acid-evoked pain being ASIC dependent, [45,110], but not all studies support these findings [111]. Experimentally, acute application of acid is associated with certain limitations, for example, not being sure of what pH nerve terminals actually encounter and how acute acid application corresponds to the acid stimulation that nociceptors encounter under pathological conditions. Moreover, pharmacological targeting of ASICs for the treatment of pain is complicated by the fact that ASICs are expressed throughout the mammalian nervous system [75] as homo- and heterotrimers and they are implicated in many physiological processes, e.g. mechanosensation [112], proprioception [113] and synaptic plasticity [114].

In summary, ASICs are proton-sensitive in a wide range of phyla with more distantly related ion channels being activated by peptides, whereas ASICs undergo peptide modulation. Alongside roles in normal neurophysiology, ASICs play a key role in inflammatory pain in mammals, which warrants further investigation as potential sites for therapeutic intervention.

## 3. Transient receptor potential ion channels

The transient receptor potential (*trp*) gene was first identified from a *Drosophila melanogaster* mutant which exhibited insensitivity to light despite retaining normal eye structure [115], the protein product of this gene was later shown to be a cationic ion channel [116]. Several homologous genes have since been identified in *D. melanogaster*, leading to the emergence of the TRPs as an independent family of ion channels comprising several subgroups [117]. TRP channels appear to have emerged before the divergence of fungi and animals, with transient receptor potential polycystin (TRPP) and transient receptor potential vanilloid (TRPV) homologues being identified in the protist *Thecamonas trahens* and ancestral genes representing five of the mammalian TRP subfamilies arising by the speciation of choanoflagellates [118]. Given TRPs can be traced back to unicellular eukaryotes, and their common expression at the plasma membrane, they probably evolved as sensors of the extracellular environment. Over evolutionary time, the number and diversity of TRP channels has increased: a total of 13 TRP genes have been discovered for *D. melanogaster*, *C. elegans*' genome contains 17, there are 28 mouse TRPs and 27 human TRPs have been identified. TRPs are generally considered to function as homotetramers, each subunit comprises intracellular N- and C-termini and six transmembrane domains, a re-entrant loop between the fifth and sixth transmembrane domains forms the channel pore [119]; it should be noted that there is however recent evidence of heteromeric TRP channel configurations [120]. Variations in motifs and modalities present within the receptors permit them to respond to a diverse range of stimuli including both chemicals and physical properties such as pressure, light and temperature. Additionally, TRPs are heavily influenced by levels of plasma membrane phospholipids and exhibit extensive phosphoregulation, enabling integration of external and internal signals. Given the sensitivity of TRPs to numerous stimuli it is perhaps unsurprising that many are expressed throughout the nervous system, in particular by sensory neurons where they confer a high degree of polymodality [121].

The ability of TRPs to respond to protons was first demonstrated for TRPV1: acidic pH potentiating capsaicin-induced inward currents [122] and protons later being shown to directly activate TRPV1 [123]. Since this finding other TRPs have been shown to be activated or positively modulated by extracellular pH, including TRPV4 [124], transient receptor potential melastatin (TRPM)7 [125], TRPP2, TRPP3 [126], transient receptor potential canonical (TRPC)4, TRPC5 [127] and transient receptor potential ankyrin (TRPA)1 [128]. There is evidence that all of these TRPs are expressed in nociceptors, albeit at differing expression levels [22,23], and whereas some TRPs are inhibited by extracellular acidosis [127] or activated by intracellular acidosis [129], in most instances proton-induced TRP activation on nociceptors results in cation influx, depolarization and nociceptor activation. While the ability of most of these TRPs to respond to protons was discovered with rodent variants of the receptors, interestingly, only the human variant of TRPA1 (hTRPA1) displays proton sensitivity. hTRPA1 was found to be active within an extracellular pH range of 7.0–5.4, while the closely related Rhesus monkey (*Macaca mulatta*) TRPA1, which shares 98% sequence homology with hTRPA1, was shown to be proton-insensitive. Comparisons of the primary sequences of the two channels identified four non-conserved amino acids distributed around the start of the sixth transmembrane domain, which when mutated reduced hTRPA1 proton sensitivity [128]. Molecular studies of TRPV1 have also pinpointed residues around transmembrane domain six as conferring proton sensitivity independently of capsaicin- and heat-sensitivity [130]. Similarly, the mutation of glutamate residues present in the re-entrant loop of TRPC5 abolishes acid-induced activation [127]. Although the specific residues differ, the importance of the re-entrant loop and transmembrane domain six in conferring proton sensitivity of TRPs is evident (figure 1). Taken together, the fact that the residues important for proton sensitivity are not conserved and the finding that only hTRPA1 is proton-sensitive, it is likely that the ability of TRPs to respond to protons evolved separately within each subfamily, highlighting the evolutionary importance of acid-sensation.

Avoidance of acidic environments by *C. elegans* suggests an ability of the nematode to detect extracellular pH and avoid acidic areas. This has been shown to be mediated by *osm-9*, a proposed homologue of mammalian TRPV channels, as reduced acid-avoidance behaviour was observed in *osm-9* mutants and following treatment of wild-type nematodes with the broad-spectrum TRP inhibitor ruthenium red [33]. Evidence explicitly linking proton-induced TRP signalling as contributing to the manifestation of pain is relatively scarce for higher-order organisms, perhaps owing to the promiscuous nature of TRP activation, and there is conflicting evidence for an involvement of TRPV1 in acute acid-induced nociception in humans [110,111]; however, a large body of evidence implicates TRPs in thermal hyperalgesia and the role of TRPs in pain has been comprehensively reviewed [131]. Indeed, studies of knockout mice suggest that both TRPV1 and ASIC3 are relatively redundant in the development of acute pain, but significantly contribute to hypersensitivity [132]. Given the prominence of acidosis in many conditions associated with pain, particularly at chronic stages following influx of immune cells, the establishment of a hypoxic environment [133,134], and the high expression of proton-sensitive TRPs by nociceptors [135] it is widely accepted that acidosis probably potentiates TRPs resulting in hyperalgesia, something well supported by *in vitro* evidence with substantiation needed *in vivo* [123,127,128]. In addition to TRP potentiation priming nociceptors, leading to more frequent action potential discharge, activation of TRPs in sensory neurons has been shown to coordinate release of the neuropeptides substance P and calcitonin gene-related peptide, which can in turn prime other neurons leading to hyperalgesia as well as contribute to central sensitization [136–138].

To summarize, TRP channels are clearly implicated in nociception, but the promiscuous nature of these receptors makes it difficult to specifically attribute proton-activation as causing TRP-mediated nociception. However, given the correlation between localized acidosis and inflammation it is likely proton-induced TRP-signalling is important in the manifestation of inflammatory pain.

## 4. Two-pore potassium channels

The two-pore (K2P) domain ion channel family comprises membrane proteins, encoded by the *kcnk* genes, that share a common molecular architecture, consisting of four transmembrane domains (TM1-4), two pore-forming domains (P1 and P2) and an extracellular cap between the TM1 and the P1 domains, assembling as either homo- or heterodimers (figure 1) [139–141]. K2P channels underlie the background K^+^ current observed in excitable and non-excitable cells, playing a key role in setting the resting membrane potential and input resistance in neurons, therefore regulating cellular excitability [142,143]. Additionally, K2P channel activity is influenced by many physico-chemical factors including extra- and intracellular pH, temperature, membrane stretch, as well as being modulated by membrane lipids and volatile anaesthetics, i.e. like ASICs and TRP channels, K2P channels integrate a number of external and internal signals. In mammals, 15 different K2P subunits have been identified and grouped into six different subclasses (TWIK, TREK, TASK, TALK, THIK and TRESK) based on their sequence similarity and functional properties [142]; transcript processing and post-translational modifications further increase their diversity [144,145]. Nevertheless, K2P channels are not restricted to mammals, being highly conserved during evolution. The first ion channel presenting two pore-forming domains per subunit was identified in the yeast *Saccharomyces cerevisiae* and named TOK1 (YORK), however, this channel differs from the mammalian K2P channels by having eight TMs [146], rather than the four observed in mammalian K2Ps. Furthermore, K2P channels with a 2P/4TM architecture have been identified in a range of different animal species, including: the marine sponge *A. queenslandica* [147], the marine opisthobranch *Aplysia californica* [148], *D. rerio* [149], *D. melanogaster* [150], *C. elegans* [151], *M. musculus* [152], *R. norvegicus* [153] and *H. sapiens* [139], i.e. K2P channels are an ancient ion channel family.

Most mammalian K2P channels are modulated by extra- and intracellular acidification. In particular, the inhibition of proton-sensitive K2Ps by extracellular acidic pH reduces constitutive K^+^ efflux produced by these channels, thus contributing to membrane depolarization and acid-induced nociception. Within the K2P channel family, TASK1 and TASK3 channels from human, mouse, rat and guinea pig (TASK3) [153–156] are markedly sensitive to extracellular acidification. The protonation of H98 (figure 1), immediately following the K^+^ selectivity filter sequence (GYG) in the P1 loop, primarily confers proton sensitivity to these channels [155–157], although mutation of this residue does not completely abolish proton sensitivity and the participation of other residues in the extracellular domain (H72 and K210: mTASK1 numbering) has also been demonstrated [158]. Moreover, TASK1 and TASK3 can form heteromeric channels, presenting intermediate properties in terms of proton sensitivity [159]. In *D. melanogaster*, 10 putative K2P channels have been identified by homology screening, but only two of them share a significant sequence identity with mammalian K2P. dTASK6 (52–57% identity with hTASKs [160]), appears to be proton-sensitive, however, mutation of conserved histidines (H98 and H72 in hTASKs) does not produce proton-insensitive channels, and it has been suggested that other residues in the M1-P1 loop are involved in this process [161]. Moreover, dTASK7 (49–55% identity with hTASKs [160]) does not form homomeric functional channels owing to two non-conserved residues (A92 and M93) in the P1 domain and although mutation of these residues to conserved threonines produces functional channels, they are still proton-insensitive, even though dTASK7 presents the conserved histidines (H98 and H72) in its sequence [161], reaffirming the involvement of other residues/regions in the proton sensitivity of these channels. In *C. elegans*, 47 genes encode K2P channels [160,162,163]. Among them, SUP-9 and TWK-20 exhibit significant sequence identity with hTASKs (43–57%) [160], however, their proton sensitivity has not yet been tested. Members of the TALK family are mainly activated in the alkaline pH range; however, they are markedly inhibited by protons, being less active (TASK2), largely inhibited (TALK1) or completely inhibited (TALK2) at pH 7.4 [164,165]. Similarly, zebrafish TASK2 (zTASK2) displays a comparable proton sensitivity profile as its mouse homologue [166]. Human TWIK1 channels are also inhibited by extracellular acidosis owing to the protonation of a homologous histidine (H122) in the P1 domain [167], nonetheless, TWIK1 produce only a very small current in heterologous systems and native conditions owing to post-translational modifications (sumoylation) [167,168]. In addition, it has been shown that during extracellular acidification, TWIK1, TASK1 and TASK3 become permeable to Na^+^ [169,170], a trait also observed in TWIK1 in hypokalaemic conditions [171]. TRESK, another member of the K2P family regulated by intracellular Ca^2+^ [172], is also sensitive to extracellular acidosis, however, whereas mouse/rat TRESK are inhibited by extracellular low pH because of a homologous histidine (H132 in mTRESK), the presence of tyrosine in the same position makes hTRESK proton-insensitive [173,174]; an arginine residue in the zebrafish TRESK homologue might result in proton-insensitivity, but this has not been tested [175]. With regard to TREKs, mutation of a different histidine in the M1P1 extracellular loop of murine TREK1 (H126), TREK2 (H151) and TRAAK (H85) channels demonstrated its involvement in their pH sensitivity. However, whereas TREK1 and TRAAK are inhibited by extracellular acidification, TREK2 is activated, this differential pH modulation involved other charged residues in the P2M4 domain [176]. Moreover, both TREK1 and TREK2 are activated by intracellular acidification, with residues in the C-terminus responsible for this activation [177,178]. Interestingly, another study showed that the proton sensitivity of hTREK1 follows a different mechanism (C-type inactivation), involving two different surfaced-exposed histidines (H87 and H141; not conserved in mTREK1 and TREK2) in the turret loop [179]. Altogether, K2P channel proton sensitivity is common to most subfamilies, involving protonatable histidine residues in the extracellular domain (figure 1), a trait conserved across mammalian species and in *D. melanogaster*, however, the lack of functional data with regard to the proton sensitivity of K2P channels in other species, including model organisms such as *C. elegans*, prevents a more extensive evolutionary analysis of K2P channel proton sensitivity.

When considering physiological roles of K2P channels, their key role in setting neuronal excitability has resulted in attempts to elucidate their participation in nociception [143,180], largely through manipulation of gene expression owing to the lack of specific K2P channel agonists and antagonists. For instance, TASK1^−/−^ and TASK3^−/−^ mice have altered thermal perception, whereas TASK1^−/−^ mice display an increased sensitivity to hot temperatures [181], TASK3^−/−^ mice are hypersensitive to cold temperatures [182], suggesting roles for both channels in thermosensation. Moreover, TASK3 is enriched in TRPM8-positive cold-sensitive neurons, which display a decreased thermal threshold after TASK3 silencing and in neurons from TASK3^−/−^ mice [182], and expression of TASK3 in Na_V_1.8-negative neurons has been shown to be involved in innocuous and acute noxious cooling [183]. During inflammation, the mRNA levels of TASK1 and TASK3 are reduced and this reduction has been correlated to spontaneous pain behaviours [184]. Furthermore, after spared sciatic nerve injury, TASK3 and TWIK1 are downregulated in lumbar 4 and 5 DRG, while TASK1 expression remains constant, however, TASK3 expressions return to baseline levels in weeks, whereas downregulation of TWIK1 persisted for months [185]. In a similar fashion, TRESK, the most highly expressed K2P channel in DRG neurons, which together with TREK2 mediates most of the background K^+^ current in small- and medium-sized DRG neurons, i.e. likely nociceptors [173], is downregulated in inflammation partially underlying spontaneous pain behaviour observed in rats [184]. In addition, it has been shown rat TRESK (rTRESK) is inhibited by arachidonic acid and hypertonic medium [174,186], as well as protons, all mediators found in the inflammatory soup, and these effects appear to be additive [174], highlighting the role of TRESK on neuronal excitability during inflammation. Corroborating the idea of TRESK being important in sensory neuronal excitability, overexpression of TRESK in trigeminal neurons reduces neuronal excitability [187] and TRESK overexpression in DRG and spinal cord after nerve injury alleviates neuropathic pain in rats [188]. Moreover, analysis of migraine genetics supports a role for TRESK. The dominant negative mutation F139WfsX24 (TRESK-MT) downregulates TRESK wild-type channels, inducing hypersensitivity of trigeminal neurons [189] and occurs in patients experiencing familial migraine with aura [190]. Intriguingly, a further TRESK mutation (C110R), found in control and migraine patients [191] that produces a complete loss of TRESK function, does not however induce trigeminal neuron hyperexcitability [192]. A recent study has described a novel transcriptional mechanism, frameshift mutation-induced alternative translation initiation (fsATI), that resolves the role of TRESK. During TRESK-MT transcription, fsATI leads to the production of a second protein fragment (MT2) that inhibits TREK1/TREK2 activity increasing sensory neuronal excitability in trigeminal neurons, i.e. both non-functional TRESK-MT and inhibition of TREK1/TREK2 are required to induce migraine-like pain states in mice [193]. Lastly, members of the TREK channel family are modulated by a wide range of physico-chemical factors, including temperature and mechanical stretch, and they have been implicated in polymodal pain perception. TREK1^−/−^ mice are more sensitive to painful heat near the threshold between warmth and noxious heat, exhibit greater mechanical sensitivity and enhanced inflammatory hypersensitivity, suggesting a role for TREK1 in peripheral nociceptor sensitization in inflammation [194]. Moreover, extracellular acidosis and lysophosphatidic acid, two inflammatory mediators, inhibit TREK1 activity [195]. TREK2^−/−^ mice show enhanced sensitivity to warmth and cool temperatures and reduced mechanical threshold in normal conditions and additionally display an absence of nocifensive behaviours in response to hypertonic saline injections after PGE_2_ sensitization [196].

In summary, the role of K2P in different aspects of nociception and pain pathophysiological states has been clearly confirmed by the use of animal models, however, their specific role in acid nociception has yet to be tested.

## 5. Proton-sensing G protein-coupled receptors

Rather than binding complex extracellular ligands, proton-sensing G protein-coupled receptors (PS-GPCRs) engage heterotrimeric G-proteins in response to mild increments in the extracellular proton concentration. To date, six mammalian PS-GPCRs have been identified: the proton sensitivity of GPR68 was described first, quickly followed by GPR4, GPR65 and GPR132, which were studied owing to high sequence similarity [197–199]. More recently, GPR31 and GPR151 were also shown to respond to extracellular protons [200]. Homologous genes for PS-GPCRs have been identified across vertebrate subphyla, all of which exhibit strong conservation of histidine residues in extracellular portions of the receptor, amino acids that confer proton sensitivity (figure 1) [197–199,201]. However, while GPR68 cloned from *D. rerio*, *X. laevis*, *G. gallus*, *M. musculus*, *R. norvegicus and H. sapiens* have all been shown to respond to protons [202], the proton sensitivity of GPR65 is not conserved across phyla. *Homo sapiens*, *M. musculus* as well as *X. laevis* GPR65 all respond to extracellular acidification while the *G. gallus* and *D. rerio* homologues do not [203]. It is thus apparent that the selective pressure for individual receptors to respond to protons is not as strong as that for expression of PS-GPCRs in general. It can thus be postulated that there may be redundancy across the PS-GPCRs, which may also offer some explanation to the lack of a strong phenotype shown by individual knock-out mice lines. Whether or not the PS-GPCRs shown to be proton-insensitive respond to other agonists or confer physiological roles remains to be determined.

PS-GPCRs exhibit widespread tissue distribution [204] and are expressed in mammalian sensory neurons [22,23], thus they probably evolved as sensors of the extracellular environment, being engaged in response to local pH perturbations and functioning to maintain homeostasis. This has been reaffirmed by studies of knockout mice, which are in the most part phenotypically normal [205], however issues regarding autoimmunity have been reported, implicating these receptors in immune cell function [206,207]. Many PS-GPCRs have also been shown to respond to various lipids, such as GPR65 which responds to psychosine [208]. Whether the receptors first arose as proton sensors or receptors for lipids is unknown, but may be addressed by studying evolutionary older variants. PS-GPCRs are expressed throughout the central and peripheral nervous systems. Importantly, a high degree of co-expression with peripherin and TRPV1, both markers of small-diameter nociceptors has been observed [204], suggesting a role of these receptors in acid nociception. Further to this, expression of GPR4, GPR65 and GPR132 has been shown to be upregulated at the transcriptional level in various rodent models of inflammation [209]. Indeed, a body of evidence exploring the roles of PS-GPCRs in inflammation has started to amass in recent years.

Taking each PS-GPCR in turn, GPR4 preferentially couples to Gα_s_ proteins following acidic challenge, resulting in the accumulation of cAMP, half maximal activation of this pathway occurs in response to pH 7.55 in HEK293 cells transiently expressing GPR4 [197]. Although unlikely to result in acute nociceptor activation, downstream signalling from Gα_s_ plays a key role in nociceptor sensitization [210,211], i.e. proton-induced activation of GPR4 can sensitize nociceptor function, which may be important to the sensitization process during chronic pathological conditions associated with tissue acidosis. Elevated GPR4 mRNA has been detected in a murine model of inflammatory pain [209], as well as in the colon and intestinal tissues of human ulcerative colitis and Crohn's disease patients. Moreover, GPR4^−/−^ mice responded less severely to a colitis model in terms of weight loss, histological damage and leucocyte infiltration [212,213]. Accordingly, acid-induced activation of GPR4 coordinates increased expression of pro-inflammatory genes and enhances immune cell recruitment following acidosis [214]. Miltz and colleagues have recently developed a novel antagonist of GPR4, which as well as preventing proton-induced cAMP accumulation in cell lines, was found to be orally active and could reduce swelling and prevent joint damage in an arthritis model as well as reduce mechanical hyperalgesia following complete Freund's adjuvant (CFA)-induced inflammation [215].

GPR65, also referred to as the T-cell death-associated gene 8 (TDAG8) receptor, is activated by protons, as well as the glycosphingolipid psychosine and the synthetic compound BTB09089. While protons and BTB09089 cause GPR65-mediated accumulation of cAMP [198,216] and thus could lead to nociceptor sensitization as described above, psychosine inhibits adenylate cyclase and mobilizes intracellular Ca^2+^ [208], suggesting an inherent G-protein bias; mobilization of intracellular Ca^2+^ both depolarizes neurons and activates PKC and thus psychosine-mediated GPR65 activation could both activate and sensitize nociceptors. Genome-wide association studies have reported correlation between single nucleotide polymorphisms in GPR65 and the inflammatory conditions chronic obstructive pulmonary disease-asthma overlap and ankylosing spondylitis [217,218]. GPR65^−/−^ mice are more susceptible to developing colitis, which has been linked to an influence of GPR65 on lysosomal function and pathogen clearance [219], however, no link between the gene and inflammatory bowel disease was observed in a Chinese population [220]. Neuronal expression of GPR65 has been shown to increase in murine models of bone cancer pain and following carrageenan- or CFA-induced inflammation, and when siRNA targeting GPR65 is administered to animals before the induction of these models less mechanical hyperalgesia is observed [221,222]. Direct activation of GPR65 with BTB09089 also produces mechanical allodynia [222]. Studies of cultured DRG and HEK293T cells have also shown that GPR65 is able to enhance capsaicin-induced Ca^2+^ fluxes through TRPV1, indicative of a pro-inflammatory role of the receptor [209,223]. A study of TRPV1, ASIC3 and GPR65 knockout animals has shown that while all three receptors are important for the manifestation of chronic inflammation following injection of CFA, only loss of GPR65 prevented the acute phase, suggesting that different acid sensors play different roles in inflammatory hyperalgesia [132]. Despite the associations between GPR65 and inflammatory conditions, studies of GPR65 and immune cells have reported decreases in the production of pro-inflammatory cytokines and upregulation of protective factors, following proton-induced activation [216,224,225]. Similarly, the expression of pro-inflammatory cytokines was elevated in GPR65^−/−^ mice compared to wild-types in a colitis model, the same was observed for a T-cell transfer colitis model when the T-cells were harvested from GPR65^−/−^ mice, however, the differential expression of inflammatory mediators between GPR65^−/−^ and wild-type animals did not ameliorate the disease pathology [226]. Taken together there appears to be a paradox surrounding the role of GPR65 in inflammation with the role of GPR65 as pro- or anti-inflammatory being highly dependent on the cellular context. GPR65 thus represents an interesting receptor for studying the neuroimmune axis of inflammation.

GPR68, or the ovarian cancer G protein-coupled receptor 1 (OGR1), so called as it was first cloned from an ovarian cancer cell line [227], stimulates the accumulation of inositol phosphates and mobilization of intracellular Ca^2+^ in response to extracellular acidosis within the range of pH 7.6–6.8 [228]. This suggests a Gα_q_ coupling, which would both directly activate nociceptors (through Ca^2+^ mobilization and depolarization) and coordinate sensitization (through PKC activation). More recently, GPR68 has also been shown to be activated by benzodiazepines and physical stress [229,230]. GPR68 has been implicated in the production of pro-inflammatory cytokines by a number of cell types in response to extracellular acidification, including pancreatic β-cells, osteoblasts and aortic smooth muscle cells [231–233]. Fittingly, GPR68 has been postulated to be the molecular mediator behind asthma-associated inflammation owing to its coordinating role in the production of interleukin-6 in response to bronchial acidosis [234]. Additionally, GPR68 has been highlighted as a potential target in the treatment of heartburn associated pain given the high expression of this receptor compared to other PS-GPCRs within oesophageal C-fibres [235]. Hypoxia has been reported to contribute to increased expression of GPR68 by intestinal macrophages and colonic tissue [236] and further to this, elevated GPR68 mRNA was observed in intestinal mucosa from ulcerative colitis and Crohn's disease patients [237]. Studies of GPR68^−/−^ mice have identified several genes whose expression is dependent on activation of GPR68 by extracellular acidosis. Loss of GPR68 was also shown to be protective in a murine model of spontaneous colitis, with less inflammation and myeloperoxidase activity as well as a lower incidence of colonic prolapse being observed [237].

GPR132, also referred to as G2A, shows more restricted expression than other PS-GPCRs and is comparatively less studied. Following acidic challenge, cells expressing GPR132 can activate Rho GTPases, however minimal activity in cAMP and inositol phosphate accumulation assays has been reported [199,238] and thus proton-induced GPR132 activation has the potential to both activate and sensitize nociceptors. Enhanced Ca^2+^ signals in response to acid challenge have been described for cells co-expressing GPR132 and GPR68, suggesting the PS-GPCRs may form oligomers to increase signalling diversity [239]. In a neuropathic pain model, G2A^−/−^ mice exhibit less mechanical hypersensitivity, however oxaliplatin, the drug used to manifest the model, was shown to increase levels of oxidized lipids which could sensitize TRPV1 via G2A and PKC dependent mechanisms [240]. By contrast, overexpression of G2A in mice reduced mechanical hypersensitivity following CFA-induced inflammation, while knockdown prolonged hyperalgesia [241]. Given that we have recently reported that the CFA model does not result in acidosis, this suggests protons are not agonizing G2A to confer the observed pain relief [55]. The role of G2A in acid nociception thus remains elusive.

Given the proton sensitivities of GPR31 and GPR151 have only recently been described, little is known about their physiological and pathophysiological roles as proton sensors. However, following spinal nerve ligation, a common model of chronic neuropathic pain, expression of GPR151 by sensory neurons was among the most upregulated genes [242], strengthening the notion of an involvement of PS-GPCRs in nociception.

While less is known about the nociceptive roles of PS-GPCRs compared to the other proton-sensitive receptors, the associations between the genes encoding these receptors and conditions associated with pain, as well as observations that loss of PS-GPCRs leads to reduced pain phenotypes in various animal models, shows that PS-GPCRs are rightfully of considerable interest in understanding the molecular mechanisms underpinning nociception.

## 6. Other acid sensors

In this review, we have focused on the main proton sensors with regard to nociceptor function. However, numerous ion channels are modulated by pH that we have not discussed here, such as inhibition of N-methyl-d-aspartate receptors, voltage-gated Ca^2+^ channels, the voltage-gated proton channel H_V_1 and ionotropic purinergic receptors but these have been reviewed elsewhere [97,243].

## 7. Integrating nociceptor acid sensitivity

As mentioned earlier, the naked mole-rat does not respond to acid as a noxious stimulus, despite a comparable expression pattern of ASICs to mice [75], functional ASIC-like and TRPV1-like currents being recorded from isolated naked mole-rat sensory neurons [58,74] and similar proton-sensitivities of cloned mice and naked mole-rat ASICs and TRPV1 [58], with the exception of ASIC3 [74]; the proton-sensing properties of K2Ps and PS-GPCRs remain unknown in this species. Recent RNA-sequencing analysis has also demonstrated that proton-sensitive ASIC3 and TWIK1 (as well as sepiapterin reductase) are both commonly downregulated in the naked mole-rat and the other proton-insensitive rodents, the Cape mole-rat and East African root rat [47]. Downstream of proton-detection, the transducer channel has to mediate sufficient depolarization to reach the activation threshold for Na_V_ subunits to initiate an action potential. However, protons can negatively regulate Na_V_ subunits, blocking the channel pore and altering the voltage-sensor movement that changes gating, as has been extensively reviewed [244], and thus Na_V_ modulation can also play a role in nociceptor acid-sensitivity. In naked mole-rat DRG neurons, macroscopic voltage-gated inward currents are significantly more susceptible to proton inhibition than those in mouse DRG neurons [58], attributed to a difference in the amino acids involved in proton inhibition, EKE replacing the relatively conserved KKV, and thus enhancing proton block. Swapping KKV for EKE in human Na_V_1.7 enhanced the degree of proton block and thus acid acts like an anaesthetic in naked mole-rat acid-sensing nociceptors to prevent action potential firing [58]. This EKE motif is also found in the proton-insensitive Cape mole-rat, whereas EKD (which has a similar -+- charge constellation) is present in other, proton-sensitive mole-rats, suggesting that additional factors are probably involved in determining proton-induced nociceptor excitability, and further negative charges in Na_V_1.7 have been identified as common only to the naked and Cape mole-rats [47]. The selection of Na_V_1.7 variants probably results from the evolutionary pressure of living in a hypercapnic, but relatively safe, environment and thus the change in Na_V_1.7 may represent an adaptation to prevent somatic nociceptor activation by hypercapnia-induced acidosis. Indeed, computational analysis has identified evidence of convergent evolution in the Na_V_1.7 amino acid variation associated with naked mole-rat acid-insensitivity in hibernating (but not closely related, non-hibernating) species [245] such that selection for this motif has occurred at least six times independently, i.e. the change in the Na_V_1.7 sequence may represent a form of convergent evolution to enable resistance to acid-induced nociceptor activation in species living in hypercapnic environments. However, the proton-insensitive East Africa root rat has Na_V_1.7 motifs common to proton-sensitive rodents, suggesting that in addition to selective pressure on Na_V_1.7 that there are further, divergent mechanisms responsible for proton-insensitivity in this species [47]. It should also be noted that acid nociception has usually been measured in response to subcutaneous acid administration, but responses observed are perhaps unrepresentative of a whole organism's proton sensitivity. For example, in the naked mole-rat, subcutaneous acid administration fails to induce nociceptive behaviour and skin-innervating nociceptors are proton-insensitive [42] and yet in the same species, sensory nerves innervating the distal colon are both activated and sensitized by acid (whether this correlates with visceral acid nociception is unknown) [246]. This finding correlates with the fact that while Na_V_1.7 is vital for somatic pain, it is not required for visceral pain in mice [247]. Therefore, it might well be that divergent evolutionary pressures have led to differential proton sensitivity within a single species, in the case of the naked mole-rat, life in a safe, but hypercapnic environment has resulted in loss of somatic proton-induced nociception, but the homeostatic role of acidification in the gastrointestinal tract (e.g. pathogen elimination) has led to maintained sensory neuron proton sensitivity.

**Figure 3.**
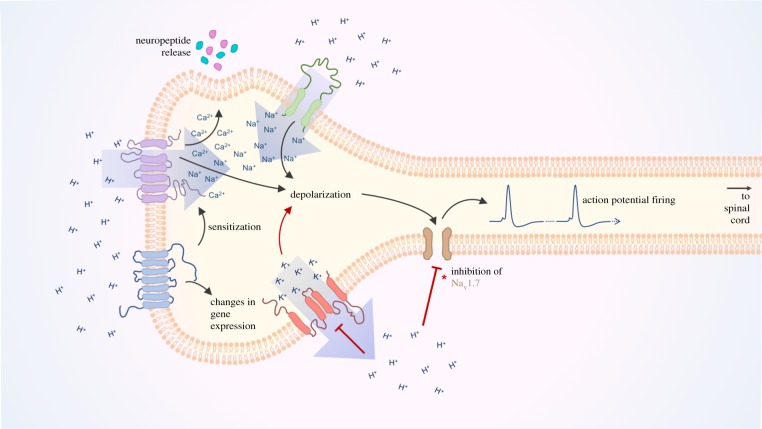
Proton-sensation at the peripheral terminal of a typical nociceptor. Following localized acidosis, the increased extracellular concentration of protons is sensed by several receptors which act in concert to increase neuronal excitability and release mediators which may sensitize other neurons. Increased extracellular proton concentration induces the activation of proton-sensitive depolarizing channels (ASICs and TRPs) causing cation influx and membrane depolarization. Simultaneously, proton-induced inhibition of K2P channels reduces constitutive K^+^ efflux further facilitating membrane depolarization. Activation of PS-GPCRs can drive changes in gene expression and coordinate phosphorylation and sensitization of TRP channels. Altogether, nociceptor membrane depolarization activates Na_V_ subunits resulting in generation of action potentials that transmit nociceptive signals to the spinal cord. (*Amino acid variations in Na_V_1.7 of some species renders the channel hypersensitive to proton-block resulting in an absence of proton-induced nociception.)

Overall, the co-expression of different proton-sensitive receptors by nociceptors allows integration of localized acidosis to a number of intracellular signalling events within nociceptors. For example, in response to a decrease in extracellular pH both ASICs and TRPs coordinate cation influx, contributing to depolarization. The permeability of TRPs to Ca^2+^ also allows them to coordinate release of neuropeptides which may act on other receptors to increase nociceptor excitability, as well as promoting inflammation. Similarly, several K2Ps expressed in nociceptors are inhibited by extracellular protons and thus constitutive K^+^ efflux is reduced, further contributing to membrane depolarization and increasing the likelihood of action potential firing. In addition to this, activation of PS-GPCRs has been shown to sensitize certain TRP channels and the role of GPCRs in transcriptional regulation may also serve to increase expression of proton-sensitive ion channels or expression of other proteins that may positively modulate nociceptor activity. The combined effect of this nociceptor priming is greater action potential discharge and heightened sensitivity to harmful stimuli, which probably manifests in pain. An overview of the integration of nociceptor acid-sensation is depicted in figure 3.

## 8. Conclusion

Detection of acid as a noxious stimulus is present in a wide variety of phyla in the Animalia kingdom, thus demonstrating that evolutionary pressure has maintained selection for this facet of nociceptor function. A wide variety of proton-sensitive receptors are expressed by sensory neurons to enable detection of the environmental pH. The range across which these different receptors are activated, as well as their modulation by a variety of other inflammatory mediators, enables sensory neurons to integrate information regarding tissue pH in both physiological and pathophysiological conditions, and the hope is that by studying the evolution of nociceptor proton sensitivity, the key molecular players can be identified, resulting in therapeutic interventions for conditions associated with tissue acidosis and pain. It is most likely this requirement of detecting a pH range that has led to the evolution of a variety of different proton-sensitive receptors, but at the same time, it is clear that many proton-sensitive receptors are modulated by other stimuli. Therefore, it could be that proton sensitivity is a remnant of a precursory role of that particular receptor, but alternatively it could be that proton sensitivity is the key role and that sensitivity to other stimuli is a feature that has remained, but with limited selection pressure, e.g. ASIC precursors are activated by peptides, whereas mammalian ASICs are proton sensors whose function is modulated by peptides.

As much as our understanding of how protons regulate nociceptors has accelerated dramatically in recent years, certain questions remain to be answered:

— many proton-sensors are activated/modulated by other stimuli, what is the physiological role of such sensors *in vivo*, detection of protons or different stimuli and what evolutionary pressures drove this dual sensitivity?
— are the ‘proton-sensitive’ residues identified in certain proton sensor families common to all proton-sensitive receptors of that family throughout evolution?
— what evolutionary pressure(s) maintained selection for the variety of acid sensors nociceptors express and how does an individual nociceptor integrate their signals? and
— what is the molecular basis of nociceptor proton sensitivity in those species where no pharmacology or genetics has yet been applied, e.g. frogs and leeches, and does this help to explain the evolution of proton-sensor function?

## Data accessibility

This article has no additional data.

## References

[1] Darwin C 1859 On the origin of species by means of natural selection, or the preservation of favoured races in the struggle for life, vol. 1 London, UK: John Murray. PMC5184128 30164232

[2] Smith ES, Lewin GR 2009 Nociceptors: a phylogenetic view. J. Comp. Physiol. A 195, 1089–1106. (10.1007/s00359-009-0482-z) PMC2780683 19830434

[3] Walters ET 2018 Nociceptive biology of molluscs and arthropods: evolutionary clues about functions and mechanisms potentially related to pain. Front. Physiol. 9, 1049 (10.3389/fphys.2018.01049) 30123137 PMC6085516

[4] Walters ET 1996 Comparative and evolutionary aspects of nociceptor function. In Neurobiology of nociceptors (eds Belmonte C, Cervero F), pp. 92–114. Oxford, UK: Oxford University Press.

[5] Sneddon LU 2018 Comparative physiology of nociception and pain. Physiology (Bethesda) 33, 63–73. 29212893 10.1152/physiol.00022.2017

[6] Burrell BD 2017 Comparative biology of pain: what invertebrates can tell us about how nociception works. J. Neurophysiol. 117, 1461–1473. (10.1152/jn.00600.2016) 28053241 PMC5376606

[7] Leys SP, Mackie GO 1997 Electrical recording from a glass sponge. Nature 387, 29–30. (10.1038/387029b0)

[8] Richards GS, Simionato E, Perron M, Adamska M, Vervoort M, Degnan BM 2008 Sponge genes provide new insight into the evolutionary origin of the neurogenic circuit. Curr. Biol. 18, 1156–1161. (10.1016/j.cub.2008.06.074) 18674909

[9] Hadrys T, DeSalle R, Sagasser S, Fischer N, Schierwater B 2005 The Trichoplax PaxB gene: a putative Proto-PaxA/B/C gene predating the origin of nerve and sensory cells. Mol. Biol. Evol. 22, 1569–1578. (10.1093/molbev/msi150) 15858210

[10] Leys SP 2015 Elements of a ‘nervous system’ in sponges. J. Exp. Biol. 218, 581–591. (10.1242/jeb.110817) 25696821

[11] Grimmelikhuijzen CJ, Westfall JA 1995 The nervous system of cnidarians. EXS 72, 7–24. 7833621 10.1007/978-3-0348-9219-3_2

[12] Passano LM, Pantin CFA 1955 Mechanical stimulation in the sea-anemone *Calliactis parasitica*. Proc. R. Soc. Lond. B 143, 226–238. (10.1098/rspb.1955.0007)

[13] Jager M, Chiori R, Alié A, Dayraud C, Quéinnec E, Manuel M 2011 New insights on ctenophore neural anatomy: immunofluorescence study in *Pleurobrachia pileus* (Müller, 1776). J. Exp. Zool. B Mol. Dev. Evol. 316B, 171–187. (10.1002/jez.b.21386) 21462312

[14] Bullock TH, Horridge GA 1965 Structure and function in the nervous system of invertebrates. San Francisco, CA: W. H. Freeman and Co.

[15] Bennett DLH, Woods CG 2014 Painful and painless channelopathies. Lancet Neurol. 13, 587–599. (10.1016/S1474-4422(14)70024-9) 24813307

[16] St John Smith E. 2018 Advances in understanding nociception and neuropathic pain. J. Neurol. 265, 231–238. (10.1007/s00415-017-8641-6) 29032407 PMC5808094

[17] Weber ES 2011 Fish analgesia: pain, stress, fear aversion, or nociception? Vet. Clin. North Am. Exot. Anim. Pract. 14, 21–32. (10.1016/j.cvex.2010.09.002) 21074700

[18] Rose J 2007 Anthropomorphism and ‘mental welfare’ of fishes. Dis. Aquat. Organ. 75, 139–154. (10.3354/dao075139) 17578253

[19] Braithwaite VA, Ebbesson LOE 2014 Pain and stress responses in farmed fish. Revue Scientifique et Technique de l'OIE 33, 245–253. (10.20506/rst.33.1.2285) 25000797

[20] Usoskin D et al. 2014 Unbiased classification of sensory neuron types by large-scale single-cell RNA sequencing. Nat. Neurosci. 18, 145–153. (10.1038/nn.3881) 25420068

[21] Li C-L et al. 2016 Somatosensory neuron types identified by high-coverage single-cell RNA-sequencing and functional heterogeneity. Cell Res. 26, 83–102. (10.1038/cr.2015.149) 26691752 PMC4816136

[22] Zeisel A et al. 2018 Molecular architecture of the mouse nervous system. Cell 174, 999–1014.e22. (10.1016/j.cell.2018.06.021) 30096314 PMC6086934

[23] Hockley JRF et al. 2019 Single-cell RNAseq reveals seven classes of colonic sensory neuron. Gut 68, 633–644. (10.1136/gutjnl-2017-315631) 29483303 PMC6580772

[24] North RY et al 2019 Electrophysiological and transcriptomic correlates of neuropathic pain in human dorsal root ganglion neurons. Brain 142, 1215–1226. (10.1093/brain/awz063) 30887021 PMC6487328

[25] Moehring F, Halder P, Seal RP, Stucky CL 2018 Uncovering the cells and circuits of touch in normal and pathological settings. Neuron 100, 349–360. (10.1016/j.neuron.2018.10.019) 30359601 PMC6708582

[26] Poole K, Moroni M, Lewin GR 2015 Sensory mechanotransduction at membrane-matrix interfaces. Pflugers Arch. 467, 121–132. (10.1007/s00424-014-1563-6) 24981693 PMC4281363

[27] Murthy SE, Dubin AE, Patapoutian A 2017 Piezos thrive under pressure: mechanically activated ion channels in health and disease. Nat. Rev. Mol. Cell Biol. 18, 771–783. (10.1038/nrm.2017.92) 28974772

[28] Bokiniec P, Zampieri N, Lewin GR, Poulet JF 2018 The neural circuits of thermal perception. Curr. Opin. Neurobiol. 52, 98–106. (10.1016/j.conb.2018.04.006) 29734030 PMC6191924

[29] Jeon S, Caterina MJ 2018 Molecular basis of peripheral innocuous warmth sensitivity. In Handbook of clinical neurology (Internet), pp. 69–82. Elsevier; (cited 2 March 2019). See https://linkinghub.elsevier.com/retrieve/pii/B9780444639127000047. 10.1016/B978-0-444-63912-7.00004-7 30454610

[30] Ruffin VA, Salameh AI, Boron WF, Parker MD 2014 Intracellular pH regulation by acid-base transporters in mammalian neurons. Front. Physiol. 5, 43 (10.3389/fphys.2014.00043) 24592239 PMC3923155

[31] Chesler M 2003 Regulation and modulation of pH in the brain. Physiol. Rev. 83, 1183–1221. (10.1152/physrev.00010.2003) 14506304

[32] Soto E, Ortega-Ramírez A, Vega R 2018 Protons as messengers of intercellular communication in the nervous system. Front. Cell. Neurosci. 12, 342 (10.3389/fncel.2018.00342) 30364044 PMC6191491

[33] Sambongi Y, Takeda K, Wakabayashi T, Ueda I, Wada Y, Futai M 2000 *Caenorhabditis elegans* senses protons through amphid chemosensory neurons: proton signals elicit avoidance behavior. Neuroreport 11, 2229–2232. (10.1097/00001756-200007140-00033) 10923676

[34] Pastor J, Soria B, Belmonte C 1996 Properties of the nociceptive neurons of the leech segmental ganglion. J. Neurophysiol. 75, 2268–2279. (10.1152/jn.1996.75.6.2268) 8793740

[35] Hamamoto DT, Simone DA 2003 Characterization of cutaneous primary afferent fibers excited by acetic acid in a model of nociception in frogs. J. Neurophysiol. 90, 566–577. (10.1152/jn.00324.2003) 12750420

[36] Hamamoto DT, Forkey MW, Davis WL, Kajander KC, Simone DA 2000 The role of pH and osmolarity in evoking the acetic acid-induced wiping response in a model of nociception in frogs. Brain Res. 862, 217–229. (10.1016/S0006-8993(00)02138-7) 10799688

[37] Pezalla PD 1983 Morphine-induced analgesia and explosive motor behavior in an amphibian. Brain Res. 273, 297–305. (10.1016/0006-8993(83)90854-5) 6604562

[38] Sneddon LU 2003 The evidence for pain in fish: the use of morphine as an analgesic. Appl. Anim. Behav. Sci. 83, 153–162. (10.1016/S0168-1591(03)00113-8)

[39] Ashley PJ, Sneddon LU, McCrohan CR 2006 Properties of corneal receptors in a teleost fish. Neurosci. Lett. 410, 165–168. (10.1016/j.neulet.2006.08.047) 17101221

[40] Samways DSK, Harkins AB, Egan TM 2009 Native and recombinant ASIC1a receptors conduct negligible Ca^2+^ entry. Cell Calcium 45, 319–325. (10.1016/j.ceca.2008.12.002) 19185346 PMC2890224

[41] Konnerth A, Lux HD, Morad M 1987 Proton-induced transformation of calcium channel in chick dorsal root ganglion cells. J. Physiol. (Lond.) 386, 603–633. (10.1113/jphysiol.1987.sp016553) 2445970 PMC1192481

[42] Park TJ et al. 2008 Selective inflammatory pain insensitivity in the African naked mole-rat (*Heterocephalus glaber*). PLoS Biol. 6, e13 (10.1371/journal.pbio.0060013) 18232734 PMC2214810

[43] Price MP et al. 2001 The DRASIC cation channel contributes to the detection of cutaneous touch and acid stimuli in mice. Neuron 32, 1071–1083. (10.1016/S0896-6273(01)00547-5) 11754838

[44] Deval E, Noel J, Lay N, Alloui A, Diochot S, Friend V, Jodar M, Lazdunski M, Lingueglia E 2008 ASIC3, a sensor of acidic and primary inflammatory pain. EMBO J. 27, 3047–3055. (10.1038/emboj.2008.213) 18923424 PMC2585165

[45] Jones NG, Slater R, Cadiou H, McNaughton P, McMahon SB 2004 Acid-induced pain and its modulation in humans. J. Neurosci. 24, 10 974–10 979. (10.1523/JNEUROSCI.2619-04.2004) PMC6730214 15574747

[46] Baumann TK, Burchiel KJ, Ingram SL, Martenson ME 1996 Responses of adult human dorsal root ganglion neurons in culture to capsaicin and low pH. Pain 65, 31–38. (10.1016/0304-3959(95)00145-X) 8826487

[47] Eigenbrod O et al. 2019 Rapid molecular evolution of pain insensitivity in multiple African rodents. Science 364, 852–859. (10.1126/science.aau0236) 31147513

[48] von Gaza W, Brandi B 1927 Die Beseittigung des Entzündungsschmerzes durch Gewerbsalkalisierung. Klin. Wochenschr. 6, 11–13. (10.1007/BF01736847)

[49] Cummings NA, Nordby GL 1966 Measurement of synovial fluid pH in normal and arthritic knees. Arthritis Rheum. 9, 47–56. (10.1002/art.1780090106) 4952418

[50] Falchuk KH, Goetzl EJ, Kulka JP 1970 Respiratory gases of synovial fluids. An approach to synovial tissue circulatory-metabolic imbalance in rheumatoid arthritis. Am. J. Med. 49, 223–231. (10.1016/S0002-9343(70)80078-X) 5452943

[51] Farr M, Garvey K, Bold AM, Kendall MJ, Bacon PA 1985 Significance of the hydrogen ion concentration in synovial fluid in rheumatoid arthritis. Clin. Exp. Rheumatol. 3, 99–104. 4017318

[52] Goldie I, Nachemson A 1969 Synovial pH in rheumatoid knee-joints. I. The effect of synovectomy. Acta Orthop. Scand. 40, 634–641. (10.3109/17453676908989529) 5378127

[53] Scholz DJ, Janich MA, Köllisch U, Schulte RF, Ardenkjaer-Larsen JH, Frank A, Haase A, Schwaiger M, Menzel MI 2015 Quantified pH imaging with hyperpolarized ^13^C-bicarbonate. Magn. Reson. Med. 73, 2274–2282. (10.1002/mrm.25357) 25046867

[54] Fujii W et al. 2015 Monocarboxylate transporter 4, associated with the acidification of synovial fluid, is a novel therapeutic target for inflammatory arthritis: MCT4 is a therapeutic target for inflammatory arthritis. Arthritis Rheumatol. 67, 2888–2896. (10.1002/art.39270) 26213210

[55] Wright AJ, Husson ZMA, Hu D-E, Callejo G, Brindle KM, Smith E 2018 Increased hyperpolarized [1-^13^C] lactate production in a model of joint inflammation is not accompanied by tissue acidosis as assessed using hyperpolarized ^13^C-labelled bicarbonate. NMR Biomed. 31, e3892 (10.1002/nbm.3892) 29380927 PMC5887936

[56] Krishtal OA, Pidoplichko VI 1980 A receptor for protons in the nerve cell membrane. Neuroscience 5, 2325–2327. (10.1016/0306-4522(80)90149-9) 6970348

[57] Smith ES, Cadiou H, McNaughton PA 2007 Arachidonic acid potentiates acid-sensing ion channels in rat sensory neurons by a direct action. Neuroscience 145, 686–698. (10.1016/j.neuroscience.2006.12.024) 17258862

[58] Smith E, Omerbašić D, Lechner SG, Anirudhan G, Lapatsina L, Lewin GR 2011 The molecular basis of acid insensitivity in the African naked mole-rat. Science 334, 1557–1560. (10.1126/science.1213760) 22174253

[59] Petruska JC, Napaporn J, Johnson RD, Gu JG, Cooper BY 2000 Subclassified acutely dissociated cells of rat DRG: histochemistry and patterns of capsaicin-, proton-, and ATP-activated currents. J. Neurophysiol. 84, 2365–2379. (10.1152/jn.2000.84.5.2365) 11067979

[60] Leffler A, Monter B, Koltzenburg M 2006 The role of the capsaicin receptor TRPV1 and acid-sensing ion channels (ASICS) in proton sensitivity of subpopulations of primary nociceptive neurons in rats and mice. Neuroscience 139, 699–709. (10.1016/j.neuroscience.2005.12.020) 16515841

[61] Bevan S, Yeats J 1991 Protons activate a cation conductance in a sub-population of rat dorsal root ganglion neurones. J. Physiol. 433, 145–161. (10.1113/jphysiol.1991.sp018419) 1726795 PMC1181364

[62] Schaefer L, Sakai H, Mattei M-G, Lazdunski M, Lingueglia E 2000 Molecular cloning, functional expression and chromosomal localization of an amiloride-sensitive Na^+^ channel from human small intestine. FEBS Lett. 471, 205–210. (10.1016/S0014-5793(00)01403-4) 10767424

[63] Waldmann R, Champigny G, Bassilana F, Heurteaux C, Lazdunski M 1997 A proton-gated cation channel involved in acid-sensing. Nature 386, 173–177. (10.1038/386173a0) 9062189

[64] Chen CC, England S, Akopian AN, Wood JN 1998 A sensory neuron-specific, proton-gated ion channel. Proc. Natl Acad. Sci. USA 95, 10 240–10 245. (10.1073/pnas.95.17.10240) PMC21492 9707631

[65] Bassler EL, Ngo-Anh TJ, Geisler HS, Ruppersberg JP, Grunder S 2001 Molecular and functional characterization of acid-sensing ion channel (ASIC) 1b. J. Biol. Chem. 276, 33 782–33 787. (10.1074/jbc.M104030200) 11448963

[66] Price MP, Snyder PM, Welsh MJ 1996 Cloning and expression of a novel human brain Na^+^ channel. J. Biol. Chem. 271, 7879–7882. (10.1074/jbc.271.14.7879) 8626462

[67] Lingueglia E, de Weille JR, Bassilana F, Heurteaux C, Sakai H, Waldmann R, Lazdunski M 1997 A modulatory subunit of acid sensing ion channels in brain and dorsal root ganglion cells. J. Biol. Chem. 272, 29 778–29 783. (10.1074/jbc.272.47.29778) 9368048

[68] Waldmann R, Bassilana F, de Weille J, Champigny G, Heurteaux C, Lazdunski M 1997 Molecular cloning of a non-inactivating proton-gated Na^+^ channel specific for sensory neurons. J. Biol. Chem. 272, 20 975–20 978. (10.1074/jbc.272.34.20975) 9261094

[69] Grunder S, Geissler HS, Bassler EL, Ruppersberg JP 2000 A new member of acid-sensing ion channels from pituitary gland. Neuroreport 11, 1607–1611. (10.1097/00001756-200006050-00003) 10852210

[70] Akopian AN, Chen CC, Ding Y, Cesare P, Wood JN 2000 A new member of the acid-sensing ion channel family. Neuroreport 11, 2217–2222. (10.1097/00001756-200007140-00031) 10923674

[71] Jasti J, Furukawa H, Gonzales EB, Gouaux E 2007 Structure of acid-sensing ion channel 1 at 1.9 Å resolution and low pH. Nature 449, 316–323. (10.1038/nature06163) 17882215

[72] Carnally SM, Dev HS, Stewart AP, Barrera NP, Van Bemmelen MX, Schild L, Henderson RM, Edwardson JM 2008 Direct visualization of the trimeric structure of the ASIC1a channel, using AFM imaging. Biochem. Biophys. Res. Commun. 372, 752–755. (10.1016/j.bbrc.2008.05.100) 18514062

[73] Donier E, Rugiero F, Jacob C, Wood JN 2008 Regulation of ASIC activity by ASIC4—new insights into ASIC channel function revealed by a yeast two-hybrid assay. Eur. J. Neurosci. 28, 74–86. (10.1111/j.1460-9568.2008.06282.x) 18662336

[74] Schuhmacher L-N, Callejo G, Srivats S, Smith ESJ 2018 Naked mole-rat acid-sensing ion channel 3 forms nonfunctional homomers, but functional heteromers. J. Biol. Chem. 293, 1756–1766. (10.1074/jbc.M117.807859) 29237731 PMC5798305

[75] Schuhmacher L-N, Smith ESJ 2016 Expression of acid-sensing ion channels and selection of reference genes in mouse and naked mole rat. Mol. Brain 9, 97 (10.1186/s13041-016-0279-2) 27964758 PMC5154015

[76] Paukert M, Chen X, Polleichtner G, Schindelin H, Grunder S 2008 Candidate amino acids involved in H^+^ gating of acid-sensing ion channel 1a. J. Biol. Chem. 283, 572–581. (10.1074/jbc.M706811200) 17981796

[77] Li T, Yang Y, Canessa CM 2009 Interaction of the aromatics Tyr-72/Trp-288 in the interface of the extracellular and transmembrane domains is essential for proton gating of acid-sensing ion channels. J. Biol. Chem. 284, 4689–4694. (10.1074/jbc.M805302200) 19074149 PMC2640969

[78] Hesselager M, Timmermann DB, Ahring PK 2004 pH dependency and desensitization kinetics of heterologously expressed combinations of acid-sensing ion channel subunits. J. Biol. Chem. 279, 11 006–11 015. (10.1074/jbc.M313507200) 14701823

[79] Smith ES, Zhang X, Cadiou H, McNaughton PA 2007 Proton binding sites involved in the activation of acid-sensing ion channel ASIC2a. Neurosci. Lett. 426, 12–17. (10.1016/j.neulet.2007.07.047) 17881127

[80] Liechti LA, Bernèche S, Bargeton B, Iwaszkiewicz J, Roy S, Michielin O, Kellenberger S 2010 A combined computational and functional approach identifies new residues involved in pH-dependent gating of ASIC1a. J. Biol. Chem. 285, 16 315–16 329. (10.1074/jbc.M109.092015) PMC2871499 20299463

[81] Della Vecchia MC, Rued AC, Carattino MD 2013 Gating transitions in the palm domain of ASIC1a. J. Biol. Chem. 288, 5487–5495. (10.1074/jbc.M112.441964) 23300086 PMC3581397

[82] Schuhmacher L-N, Srivats S, Smith ESJ 2015 Structural domains underlying the activation of acid-sensing ion channel 2a. Mol. Pharmacol. 87, 561–571. (10.1124/mol.114.096909) 25583083 PMC4747086

[83] Chen X, Polleichtner G, Kadurin I, Grunder S 2007 Zebrafish acid-sensing ion channel (ASIC) 4, characterization of homo- and heteromeric channels, and identification of regions important for activation by H^+^. J. Biol. Chem. 282, 30 406–30 413. (10.1074/jbc.M702229200) 17686779

[84] Baron A, Schaefer L, Lingueglia E, Champigny G, Lazdunski M 2001 Zn^2+^ and H^+^ are coactivators of acid-sensing ion channels. J. Biol. Chem. 276, 35 361–35 367. (10.1074/jbc.M105208200) 11457851

[85] Springauf A, Gründer S 2010 An acid-sensing ion channel from shark (*Squalus acanthias*) mediates transient and sustained responses to protons. J. Physiol. 588, 809–820. (10.1113/jphysiol.2009.182931) 20064854 PMC2834940

[86] Coric T, Zheng D, Gerstein M, Canessa CM 2005 Proton sensitivity of ASIC1 appeared with the rise of fishes by changes of residues in the region that follows TM1 in the ectodomain of the channel. J. Physiol. 568, 725–735. (10.1113/jphysiol.2005.087734) 16002453 PMC1464184

[87] Coric T, Passamaneck YJ, Zhang P, Di Gregorio A, Canessa CM 2008 Simple chordates exhibit a proton-independent function of acid-sensing ion channels. FASEB J. 22, 1914–1923. (10.1096/fj.07-100313) 18211956

[88] Lynagh T, Mikhaleva Y, Colding JM, Glover JC, Pless SA 2018 Acid-sensing ion channels emerged over 600 Mya and are conserved throughout the deuterostomes. Proc. Natl Acad. Sci. USA 115, 8430–8435. (10.1073/pnas.1806614115) 30061402 PMC6099870

[89] Cottrell GA, Green KA, Davies NW 1990 The neuropeptide Phe-Met-Arg-Phe-NH2 (FMRFamide) can activate a ligand-gated ion channel in Helix neurones. Pflugers Arch. 416, 612–614. (10.1007/BF00382698) 1700364

[90] Golubovic A, Kuhn A, Williamson M, Kalbacher H, Holstein TW, Grimmelikhuijzen CJ, Gründer S 2007 A peptide-gated ion channel from the freshwater polyp *Hydra*. J. Biol. Chem. 282, 35 098–35 103. (10.1074/jbc.M706849200) 17911098

[91] Askwith CC, Cheng C, Ikuma M, Benson C, Price MP, Welsh MJ 2000 Neuropeptide FF and FMRFamide potentiate acid-evoked currents from sensory neurons and proton-gated DEG/ENaC channels. Neuron 26, 133–141. (10.1016/S0896-6273(00)81144-7) 10798398

[92] Sherwood TW, Askwith CC 2009 Dynorphin opioid peptides enhance acid-sensing ion channel 1a activity and acidosis-induced neuronal death. J. Neurosci. 29, 14 371–14 380. (10.1523/JNEUROSCI.2186-09.2009) 19906984 PMC2802056

[93] Farrag M, Drobish JK, Puhl HL, Kim JS, Herold PB, Kaufman MP, Ruiz-Velasco V 2017 Endomorphins potentiate acid-sensing ion channel currents and enhance the lactic acid-mediated increase in arterial blood pressure: effects amplified in hindlimb ischaemia: endomorphin-mediated modulation of ASIC3 channel subunits. J. Physiol. 595, 7167–7183. (10.1113/JP275058) 29044528 PMC5709321

[94] Reimers C et al. 2017 Identification of a cono-RFamide from the venom of *Conus textile* that targets ASIC3 and enhances muscle pain. Proc. Natl Acad. Sci. USA 114, E3507–E3515. (10.1073/pnas.1616232114) 28396446 PMC5410773

[95] Vyvers A, Schmidt A, Wiemuth D, Gründer S 2018 Screening of 109 neuropeptides on ASICs reveals no direct agonists and dynorphin A, YFMRFamide and endomorphin-1 as modulators. Sci. Rep. 8, 18000 (10.1038/s41598-018-36125-5) 30573735 PMC6301962

[96] Yang X-N et al. 2017 The nonproton ligand of acid-sensing ion channel 3 activates mollusk-specific FaNaC channels via a mechanism independent of the native FMRFamide peptide. J. Biol. Chem. 292, 21 662–21 675. (10.1074/jbc.M117.814707) PMC5766947 29123030

[97] Boscardin E, Alijevic O, Hummler E, Frateschi S, Kellenberger S 2016 The function and regulation of acid-sensing ion channels (ASICs) and the epithelial Na^+^ channel (ENaC): IUPHAR Review 19: ASIC and ENaC nomenclature. Br. J. Pharmacol. 173, 2671–2701. (10.1111/bph.13533) 27278329 PMC4995293

[98] Rash LD 2017 Acid-sensing ion channel pharmacology, past, present, and future. Adv. Pharmacol. 79, 35–66. (10.1016/bs.apha.2017.02.001) 28528673

[99] Allen NJ, Attwell D 2002 Modulation of ASIC channels in rat cerebellar Purkinje neurons by ischaemia-related signals. J. Physiol. 543, 521–529. (10.1113/jphysiol.2002.020297) 12205186 PMC2290513

[100] Cadiou H, Studer M, Jones NG, Smith ES, Ballard A, McMahon SB, McNaughton PA 2007 Modulation of acid-sensing ion channel activity by nitric oxide. J. Neurosci. 27, 13 251–13 260. (10.1523/JNEUROSCI.2135-07.2007) PMC6673420 18045919

[101] Deval E, Salinas M, Baron A, Lingueglia E, Lazdunski M 2004 ASIC2b-dependent regulation of ASIC3, an essential acid-sensing ion channel subunit in sensory neurons via the partner protein PICK-1. J. Biol. Chem. 279, 19 531–19 539. (10.1074/jbc.M313078200) 14976185

[102] Steen KH, Reeh PW, Anton F, Handwerker HO 1992 Protons selectively induce lasting excitation and sensitization to mechanical stimulation of nociceptors in rat skin, *in vitro*. J. Neurosci. 12, 86–95. (10.1523/JNEUROSCI.12-01-00086.1992) 1309578 PMC6575698

[103] Callejo G, Castellanos A, Castany M, Gual A, Luna C, Acosta MC, Gallar J, Giblin JP, Gasull X 2015 Acid-sensing ion channels detect moderate acidifications to induce ocular pain. Pain 156, 483–495. (10.1097/01.j.pain.0000460335.49525.17) 25687542

[104] Blanchard MG, Rash LD, Kellenberger S 2012 Inhibition of voltage-gated Na^+^ currents in sensory neurones by the sea anemone toxin APETx2. Br. J. Pharmacol. 165, 2167–2177. (10.1111/j.1476-5381.2011.01674.x) 21943094 PMC3413854

[105] Deval E, Lingueglia E 2015 Acid-sensing ion channels and nociception in the peripheral and central nervous systems. Neuropharmacology 94, 49–57. (10.1016/j.neuropharm.2015.02.009) 25724084

[106] Sluka KA, Gregory NS 2015 The dichotomized role for acid sensing ion channels in musculoskeletal pain and inflammation. Neuropharmacology 94, 58–63. (10.1016/j.neuropharm.2014.12.013) 25582293 PMC4458430

[107] Mazzuca M et al. 2007 A tarantula peptide against pain via ASIC1a channels and opioid mechanisms. Nat. Neurosci. 10, 943–945. (10.1038/nn1940) 17632507

[108] Diochot S et al. 2012 Black mamba venom peptides target acid-sensing ion channels to abolish pain. Nature 490, 552–555. (10.1038/nature11494) 23034652

[109] Sluka KA, Price MP, Breese NM, Stucky CL, Wemmie JA, Welsh MJ 2003 Chronic hyperalgesia induced by repeated acid injections in muscle is abolished by the loss of ASIC3, but not ASIC1. Pain 106, 229–239. (10.1016/S0304-3959(03)00269-0) 14659506

[110] Ugawa S, Ueda T, Ishida Y, Nishigaki M, Shibata Y, Shimada S 2002 Amiloride-blockable acid-sensing ion channels are leading acid sensors expressed in human nociceptors. J. Clin. Invest. 110, 1185–1190. (10.1172/JCI0215709) 12393854 PMC150796

[111] Schwarz MG, Namer B, Reeh PW, Fischer MJM 2017 TRPA1 and TRPV1 antagonists do not inhibit human acidosis-induced pain. J. Pain 18, 526–534. (10.1016/j.jpain.2016.12.011) 28062311

[112] Omerbašić D, Schuhmacher L-N, Bernal Sierra Y-A, Smith E, Lewin GR 2015 ASICs and mammalian mechanoreceptor function. Neuropharmacology 94, 80–86. (10.1016/j.neuropharm.2014.12.007) 25528740

[113] Lee C-H, Chen C-C 2018 Roles of ASICs in nociception and proprioception. Adv. Exp. Med. Biol. 1099, 37–47. (10.1007/978-981-13-1756-9_4) 30306513

[114] Huang Y, Jiang N, Li J, Ji Y-H, Xiong Z-G, Zha X 2015 Two aspects of ASIC function: synaptic plasticity and neuronal injury. Neuropharmacology 94, 42–48. (10.1016/j.neuropharm.2014.12.010) 25582290 PMC4458418

[115] Cosens DJ, Manning A 1969 Abnormal electroretinogram from a *Drosophila* mutant. Nature 224, 285–287. (10.1038/224285a0) 5344615

[116] Suss-Toby E, Selinger Z, Minke B 1991 Lanthanum reduces the excitation efficiency in fly photoreceptors. J. Gen. Physiol. 98, 849–868. (10.1085/jgp.98.4.849) 1960531 PMC2229083

[117] Montell C 2005 *Drosophila* TRP channels. Pflugers Arch. 451, 19–28. (10.1007/s00424-005-1426-2) 15952038

[118] Cai X, Clapham DE 2012 Ancestral Ca^2+^ signaling machinery in early animal and fungal evolution. Mol. Biol. Evol. 29, 91–100. (10.1093/molbev/msr149) 21680871 PMC4037924

[119] Hellmich UA, Gaudet R 2014 Structural biology of TRP channels. Handb. Exp. Pharmacol. 223, 963–990. (10.1007/978-3-319-05161-1_10) 24961976 PMC5075240

[120] Bröker-Lai J et al. 2017 Heteromeric channels formed by TRPC1, TRPC4 and TRPC5 define hippocampal synaptic transmission and working memory. EMBO J. 36, 2770–2789. (10.15252/embj.201696369) 28790178 PMC5599800

[121] Sousa-Valente J, Andreou AP, Urban L, Nagy I 2014 Transient receptor potential ion channels in primary sensory neurons as targets for novel analgesics. Br. J. Pharmacol. 171, 2508–2527. (10.1111/bph.12532) 24283624 PMC4008996

[122] Caterina MJ, Schumacher MA, Tominaga M, Rosen TA, Levine JD, Julius D 1997 The capsaicin receptor: a heat-activated ion channel in the pain pathway. Nature 389, 816–824. (10.1038/39807) 9349813

[123] Tominaga M, Caterina MJ, Malmberg AB, Rosen TA, Gilbert H, Skinner K, Raumann BE, Basbaum AI, Julius D 1998 The cloned capsaicin receptor integrates multiple pain-producing stimuli. Neuron 21, 531–543. (10.1016/S0896-6273(00)80564-4) 9768840

[124] Suzuki M, Mizuno A, Kodaira K, Imai M 2003 Impaired pressure sensation in mice lacking TRPV4. J. Biol. Chem. 278, 22 664–22 668. (10.1074/jbc.M302561200) 12692122

[125] Jiang J, Li M, Yue L 2005 Potentiation of TRPM7 inward currents by protons. J. Gen. Physiol. 126, 137–150. (10.1085/jgp.200409185) 16009728 PMC2266571

[126] Chang RB, Waters H, Liman ER 2010 A proton current drives action potentials in genetically identified sour taste cells. Proc. Natl Acad. Sci. USA 107, 22 320–22 325. (10.1073/pnas.1013664107) 21098668 PMC3009759

[127] Semtner M, Schaefer M, Pinkenburg O, Plant TD 2007 Potentiation of TRPC5 by protons. J. Biol. Chem. 282, 33 868–33 878. (10.1074/jbc.M702577200) 17884814

[128] de la Roche J et al. 2013 The molecular basis for species-specific activation of human TRPA1 protein by protons involves poorly conserved residues within transmembrane domains 5 and 6. J. Biol. Chem. 288, 20 280–20 292. (10.1074/jbc.M113.479337) 23709225 PMC3711295

[129] Cao X, Yang F, Zheng J, Wang K 2012 Intracellular proton-mediated activation of TRPV3 channels accounts for the exfoliation effect of α-hydroxyl acids on keratinocytes. J. Biol. Chem. 287, 25 905–25 916. (10.1074/jbc.M112.364869) PMC3406675 22679014

[130] Aneiros E, Cao L, Papakosta M, Stevens EB, Phillips S, Grimm C 2011 The biophysical and molecular basis of TRPV1 proton gating. EMBO J. 30, 994–1002. (10.1038/emboj.2011.19) 21285946 PMC3061026

[131] Dai Y 2016 TRPs and pain. Semin. Immunopathol. 38, 277–291. (10.1007/s00281-015-0526-0) 26374740

[132] Hsieh W-S, Kung C-C, Huang S-L, Lin S-C, Sun W-H 2017 TDAG8, TRPV1, and ASIC3 involved in establishing hyperalgesic priming in experimental rheumatoid arthritis. Sci. Rep. 7, 8870 (10.1038/s41598-017-09200-6) 28827659 PMC5566336

[133] Harrison DK, Spence VA, Beck JS, Lowe JG, Walker WF 1986 pH changes in the dermis during the course of the tuberculin skin test. Immunology 59, 497–501. 3804375 PMC1453324

[134] Roiniotis J, Dinh H, Masendycz P, Turner A, Elsegood CL, Scholz GM, Hamilton JA 2009 Hypoxia prolongs monocyte/macrophage survival and enhanced glycolysis is associated with their maturation under aerobic conditions. J. Immunol. 182, 7974–7981. (10.4049/jimmunol.0804216) 19494322

[135] Mickle AD, Shepherd AJ, Mohapatra DP 2016 Nociceptive TRP channels: sensory detectors and transducers in multiple pain pathologies. Pharmaceuticals (Basel) 9, 72 (10.3390/ph9040072) PMC5198047 27854251

[136] Meng J, Ovsepian SV, Wang J, Pickering M, Sasse A, Aoki KR, Lawrence GW, Dolly JO 2009 Activation of TRPV1 mediates calcitonin gene-related peptide release, which excites trigeminal sensory neurons and is attenuated by a retargeted botulinum toxin with anti-nociceptive potential. J. Neurosci. 29, 4981–4992. (10.1523/JNEUROSCI.5490-08.2009) 19369567 PMC6665337

[137] Nakamura Y, Une Y, Miyano K, Abe H, Hisaoka K, Morioka N, Nakata Y 2012 Activation of transient receptor potential ankyrin 1 evokes nociception through substance P release from primary sensory neurons. J. Neurochem. 120, 1036–1047. (10.1111/j.1471-4159.2011.07628.x) 22182301

[138] Engel MA et al. 2011 TRPA1 and substance P mediate colitis in mice. Gastroenterology 141, 1346–1358. (10.1053/j.gastro.2011.07.002) 21763243

[139] Lesage F, Guillemare E, Fink M, Duprat F, Lazdunski M, Romey G, Barhanin J 1996 TWIK-1, a ubiquitous human weakly inward rectifying K^+^ channel with a novel structure. EMBO J. 15, 1004–1011. (10.1002/j.1460-2075.1996.tb00437.x) 8605869 PMC449995

[140] Miller AN, Long SB 2012 Crystal structure of the human two-pore domain potassium channel K2P1. Science 335, 432–436. (10.1126/science.1213274) 22282804

[141] Brohawn SG, del Mármol J, MacKinnon R 2012 Crystal structure of the human K2P TRAAK, a lipid- and mechano-sensitive K^+^ ion channel. Science 335, 436–441. (10.1126/science.1213808) 22282805 PMC3329120

[142] Enyedi P, Czirják G 2010 Molecular background of leak K^+^ currents: two-pore domain potassium channels. Physiol. Rev. 90, 559–605. (10.1152/physrev.00029.2009) 20393194

[143] Mathie A, Veale EL 2015 Two-pore domain potassium channels: potential therapeutic targets for the treatment of pain. Pflugers Arch. 467, 931–943. (10.1007/s00424-014-1655-3) 25420526

[144] Thomas D, Plant LD, Wilkens CM, McCrossan ZA, Goldstein SAN 2008 Alternative translation initiation in rat brain yields K_2P_2.1 potassium channels permeable to sodium. Neuron 58, 859–870. (10.1016/j.neuron.2008.04.016) 18579077 PMC2529466

[145] Plant LD, Zuniga L, Araki D, Marks JD, Goldstein SAN 2012 SUMOylation silences heterodimeric TASK potassium channels containing K2P1 subunits in cerebellar granule neurons. Sci. Signal. 5, ra84 (10.1126/scisignal.2003431) 23169818 PMC3876883

[146] Ketchum KA, Joiner WJ, Sellers AJ, Kaczmarek LK, Goldstein SA 1995 A new family of outwardly rectifying potassium channel proteins with two pore domains in tandem. Nature 376, 690–695. (10.1038/376690a0) 7651518

[147] Wells GD, Tang Q-Y, Heler R, Tompkins-MacDonald GJ, Pritchard EN, Leys SP, Logothetis DE, Boland LM 2012 A unique alkaline pH-regulated and fatty acid-activated tandem pore domain potassium channel (K_2P_) from a marine sponge. J. Exp. Biol. 215, 2435–2444. (10.1242/jeb.066233) 22723483 PMC3379850

[148] Jezzini SH, Moroz LL 2004 Identification and distribution of a two-pore domain potassium channel in the CNS of *Aplysia californica*. Brain Res. Mol. Brain Res. 127, 27–38. (10.1016/j.molbrainres.2004.05.007) 15306118

[149] Gierten J, Hassel D, Schweizer PA, Becker R, Katus HA, Thomas D 2012 Identification and functional characterization of zebrafish K_2P_10.1 (TREK2) two-pore-domain K^+^ channels. Biochim. Biophys. Acta 1818, 33–41. (10.1016/j.bbamem.2011.09.015) 21963410

[150] Goldstein SA, Price LA, Rosenthal DN, Pausch MH 1996 ORK1, a potassium-selective leak channel with two pore domains cloned from *Drosophila melanogaster* by expression in *Saccharomyces cerevisiae*. Proc. Natl Acad. Sci. USA 93, 13 256–13 261. (10.1073/pnas.93.23.13256) 8917578 PMC24080

[151] Wei A, Jegla T, Salkoff L 1996 Eight potassium channel families revealed by the *C. elegans* genome project. Neuropharmacology 35, 805–829. (10.1016/0028-3908(96)00126-8) 8938713

[152] Fink M, Duprat F, Lesage F, Reyes R, Romey G, Heurteaux C, Lazdunski M 1996 Cloning, functional expression and brain localization of a novel unconventional outward rectifier K^+^ channel. EMBO J. 15, 6854–6862. (10.1002/j.1460-2075.1996.tb01077.x) 9003761 PMC452511

[153] Leonoudakis D, Gray AT, Winegar BD, Kindler CH, Harada M, Taylor DM, Chavez RA, Forsayeth JR, Yost CS 1998 An open rectifier potassium channel with two pore domains in tandem cloned from rat cerebellum. J. Neurosci. 18, 868–877. (10.1523/JNEUROSCI.18-03-00868.1998) 9437008 PMC6792778

[154] Duprat F, Lesage F, Fink M, Reyes R, Heurteaux C, Lazdunski M 1997 TASK, a human background K^+^ channel to sense external pH variations near physiological pH. EMBO J. 16, 5464–5471. (10.1093/emboj/16.17.5464) 9312005 PMC1170177

[155] Rajan S, Wischmeyer E, Xin Liu G, Preisig-Müller R, Daut J, Karschin A, Derst C 2000 TASK-3, a novel tandem pore domain acid-sensitive K^+^ channel. An extracellular histiding as pH sensor. J. Biol. Chem. 275, 16 650–16 657. (10.1074/jbc.M000030200) 10747866

[156] Lopes CM, Gallagher PG, Buck ME, Butler MH, Goldstein SA 2000 Proton block and voltage gating are potassium-dependent in the cardiac leak channel Kcnk3. J. Biol. Chem. 275, 16 969–16 978. (10.1074/jbc.M001948200) 10748056

[157] Lopes CM, Zilberberg N, Goldstein SA 2001 Block of Kcnk3 by protons. Evidence that 2-P-domain potassium channel subunits function as homodimers. J. Biol. Chem. 276, 24 449–24 452. (10.1074/jbc.C100184200) 11358956

[158] Morton MJ, O'Connell AD, Sivaprasadarao A, Hunter M 2003 Determinants of pH sensing in the two-pore domain K^+^ channels TASK-1 and -2. Pflugers Arch. 445, 577–583. (10.1007/s00424-002-0901-2) 12634929

[159] Czirják G, Enyedi P 2002 Formation of functional heterodimers between the TASK-1 and TASK-3 two-pore domain potassium channel subunits. J. Biol. Chem. 277, 5426–5432. (10.1074/jbc.M107138200) 11733509

[160] Ben Soussia I et al. 2019 Mutation of a single residue promotes gating of vertebrate and invertebrate two-pore domain potassium channels. Nat. Commun. 10, 787 (10.1038/s41467-019-08710-3) 30770809 PMC6377628

[161] Döring F, Scholz H, Kühnlein RP, Karschin A, Wischmeyer E 2006 Novel *Drosophila* two-pore domain K^+^ channels: rescue of channel function by heteromeric assembly. Eur. J. Neurosci. 24, 2264–2274. (10.1111/j.1460-9568.2006.05102.x) 17074048

[162] Buckingham SD, Kidd JF, Law RJ, Franks CJ, Sattelle DB 2005 Structure and function of two-pore-domain K^+^ channels: contributions from genetic model organisms. Trends Pharmacol. Sci. 26, 361–367. (10.1016/j.tips.2005.05.003) 15939489

[163] Wang ZW, Kunkel MT, Wei A, Butler A, Salkoff L 1999 Genomic organization of nematode 4TM K^+^ channels. Ann. NY Acad. Sci. 868, 286–303. (10.1111/j.1749-6632.1999.tb11294.x) 10414302

[164] Reyes R, Duprat F, Lesage F, Fink M, Salinas M, Farman N, Lazdunski M 1998 Cloning and expression of a novel pH-sensitive two pore domain K^+^ channel from human kidney. J. Biol. Chem. 273, 30 863–30 869. (10.1074/jbc.273.47.30863) 9812978

[165] Kang D, Kim D 2004 Single-channel properties and pH sensitivity of two-pore domain K^+^ channels of the TALK family. Biochem. Biophys. Res. Commun. 315, 836–844. (10.1016/j.bbrc.2004.01.137) 14985088

[166] Peña-Münzenmayer G, Niemeyer MI, Sepúlveda FV, Cid LP 2014 Zebrafish and mouse TASK-2 K^+^ channels are inhibited by increased CO_2_ and intracellular acidification. Pflugers Arch. 466, 1317–1327. (10.1007/s00424-013-1365-2) 24081451

[167] Rajan S, Plant LD, Rabin ML, Butler MH, Goldstein SAN 2005 Sumoylation silences the plasma membrane leak K^+^ channel K2P1. Cell 121, 37–47. (10.1016/j.cell.2005.01.019) 15820677

[168] Plant LD, Dementieva IS, Kollewe A, Olikara S, Marks JD, Goldstein SAN 2010 One SUMO is sufficient to silence the dimeric potassium channel K2P1. Proc. Natl Acad. Sci. USA 107, 10 743–10 748. (10.1073/pnas.1004712107) PMC2890841 20498050

[169] Chatelain FC, Bichet D, Douguet D, Feliciangeli S, Bendahhou S, Reichold M, Warth R, Barhanin J, Lesage F 2012 TWIK1, a unique background channel with variable ion selectivity. Proc. Natl Acad. Sci. USA 109, 5499–5504. (10.1073/pnas.1201132109) 22431633 PMC3325654

[170] Ma L, Zhang X, Zhou M, Chen H 2012 Acid-sensitive TWIK and TASK two-pore domain potassium channels change ion selectivity and become permeable to sodium in extracellular acidification. J. Biol. Chem. 287, 37 145–37 153. (10.1074/jbc.M112.398164) PMC3481314 22948150

[171] Ma L, Zhang X, Chen H 2011 TWIK-1 two-pore domain potassium channels change ion selectivity and conduct inward leak sodium currents in hypokalemia. Sci. Signal. 4, ra37 (10.1126/scisignal.2001726) 21653227

[172] Czirják G, Tóth ZE, Enyedi P 2004 The two-pore domain K^+^ channel, TRESK, is activated by the cytoplasmic calcium signal through calcineurin. J. Biol. Chem. 279, 18 550–18 558. (10.1074/jbc.M312229200) 14981085

[173] Dobler T, Springauf A, Tovornik S, Weber M, Schmitt A, Sedlmeier R, Wischmeyer E, Döring F 2007 TRESK two-pore-domain K^+^ channels constitute a significant component of background potassium currents in murine dorsal root ganglion neurones. J. Physiol. (Lond.) 585, 867–879. (10.1113/jphysiol.2007.145649) 17962323 PMC2375503

[174] Callejo G, Giblin JP, Gasull X 2013 Modulation of TRESK background K^+^ channel by membrane stretch. PLoS ONE 8, e64471 (10.1371/journal.pone.0064471) 23691227 PMC3655163

[175] Rahm A-K, Wiedmann F, Gierten J, Schmidt C, Schweizer PA, Becker R, Katus HA, Thomas D 2014 Functional characterization of zebrafish K_2P_18.1 (TRESK) two-pore-domain K^+^ channels. Naunyn Schmiedebergs Arch. Pharmacol. 387, 291–300. (10.1007/s00210-013-0945-1) 24311292

[176] Sandoz G, Douguet D, Chatelain F, Lazdunski M, Lesage F 2009 Extracellular acidification exerts opposite actions on TREK1 and TREK2 potassium channels via a single conserved histidine residue. Proc. Natl Acad. Sci. USA 106, 14 628–14 633. (10.1073/pnas.0906267106) PMC2732798 19667202

[177] Maingret F, Patel AJ, Lesage F, Lazdunski M, Honoré E 1999 Mechano- or acid stimulation, two interactive modes of activation of the TREK-1 potassium channel. J. Biol. Chem. 274, 26 691–26 696. (10.1074/jbc.274.38.26691) 10480871

[178] Kim Y, Gnatenco C, Bang H, Kim D 2001 Localization of TREK-2 K^+^ channel domains that regulate channel kinetics and sensitivity to pressure, fatty acids and pH_i_. Pflugers Arch. 442, 952–960. (10.1007/s004240100626) 11680629

[179] Cohen A, Ben-Abu Y, Hen S, Zilberberg N 2008 A novel mechanism for human K_2P_2.1 channel gating. Facilitation of C-type gating by protonation of extracellular histidine residues. J. Biol. Chem. 283, 19 448–19 455. (10.1074/jbc.M801273200) 18474599

[180] Gada K, Plant LD 2019 Two-pore domain potassium channels: emerging targets for novel analgesic drugs: IUPHAR Review 26. Br. J. Pharmacol. 176, 256–266. (10.1111/bph.14518) 30325008 PMC6295411

[181] Linden A-M et al. 2006 The *in vivo* contributions of TASK-1-containing channels to the actions of inhalation anesthetics, the α_2_ adrenergic sedative dexmedetomidine, and cannabinoid agonists. J. Pharmacol. Exp. Ther. 317, 615–626. (10.1124/jpet.105.098525) 16397088

[182] Morenilla-Palao C, Luis E, Fernández-Peña C, Quintero E, Weaver JL, Bayliss DA, Viana F 2014 Ion channel profile of TRPM8 cold receptors reveals a role of TASK-3 potassium channels in thermosensation. Cell Rep. 8, 1571–1582. (10.1016/j.celrep.2014.08.003) 25199828 PMC5724366

[183] Luiz AP, MacDonald DI, Santana-Varela S, Millet Q, Sikandar S, Wood JN, Emery EC 2019 Cold sensing by Na_V_1.8-positive and Na_V_1.8-negative sensory neurons. Proc. Natl Acad. Sci. USA 116, 3811–3816. (10.1073/pnas.1814545116) 30755524 PMC6397562

[184] Marsh B, Acosta C, Djouhri L, Lawson SN 2012 Leak K^+^ channel mRNAs in dorsal root ganglia: relation to inflammation and spontaneous pain behaviour. Mol. Cell. Neurosci. 49, 375–386. (10.1016/j.mcn.2012.01.002) 22273507 PMC3334831

[185] Pollema-Mays SL, Centeno MV, Ashford CJ, Apkarian AV, Martina M 2013 Expression of background potassium channels in rat DRG is cell-specific and down-regulated in a neuropathic pain model. Mol. Cell. Neurosci. 57, 1–9. (10.1016/j.mcn.2013.08.002) 23994814 PMC3842394

[186] Sano Y et al. 2003 A novel two-pore domain K^+^ channel, TRESK, is localized in the spinal cord. J. Biol. Chem. 278, 27 406–27 412. (10.1074/jbc.M206810200) 12754259

[187] Guo Z, Cao Y-Q 2014 Over-expression of TRESK K^+^ channels reduces the excitability of trigeminal ganglion nociceptors. PLoS ONE 9, e87029 (10.1371/journal.pone.0087029) 24466320 PMC3900698

[188] Zhou J, Yang C-X, Zhong J-Y, Wang H-B 2013 Intrathecal TRESK gene recombinant adenovirus attenuates spared nerve injury-induced neuropathic pain in rats. Neuroreport 24, 131–136. (10.1097/WNR.0b013e32835d8431) 23370493

[189] Liu P, Xiao Z, Ren F, Guo Z, Chen Z, Zhao H, Cao Y-Q 2013 Functional analysis of a migraine-associated TRESK K^+^ channel mutation. J. Neurosci. 33, 12 810–12 824. (10.1523/JNEUROSCI.1237-13.2013) PMC3728689 23904616

[190] Lafrenière RG et al. 2010 A dominant-negative mutation in the TRESK potassium channel is linked to familial migraine with aura. Nat. Med. 16, 1157–1160. (10.1038/nm.2216) 20871611

[191] Andres-Enguix I et al. 2012 Functional analysis of missense variants in the TRESK (KCNK18) K^+^ channel. Sci. Rep. 2, 237 (10.1038/srep00237) 22355750 PMC3266952

[192] Guo Z, Liu P, Ren F, Cao Y-Q 2014 Nonmigraine-associated TRESK K^+^ channel variant C110R does not increase the excitability of trigeminal ganglion neurons. J. Neurophysiol. 112, 568–579. (10.1152/jn.00267.2014) 24805079 PMC4122697

[193] Royal P et al. 2019 Migraine-associated TRESK mutations increase neuronal excitability through alternative translation initiation and inhibition of TREK. Neuron 101, 232–245.e6. (10.1016/j.neuron.2018.11.039) 30573346

[194] Alloui A et al. 2006 TREK-1, a K^+^ channel involved in polymodal pain perception. EMBO J. 25, 2368–2376. (10.1038/sj.emboj.7601116) 16675954 PMC1478167

[195] Cohen A, Sagron R, Somech E, Segal-Hayoun Y, Zilberberg N 2009 Pain-associated signals, acidosis and lysophosphatidic acid, modulate the neuronal K_2P_2.1 channel. Mol. Cell. Neurosci. 40, 382–389. (10.1016/j.mcn.2008.12.004) 19130888

[196] Pereira V et al. 2014 Role of the TREK2 potassium channel in cold and warm thermosensation and in pain perception. Pain 155, 2534–2544. (10.1016/j.pain.2014.09.013) 25239074

[197] Ludwig M-G, Vanek M, Guerini D, Gasser JA, Jones CE, Junker U, Hofstetter H, Wolf RM, Seuwen K 2003 Proton-sensing G-protein-coupled receptors. Nature 425, 93–98. (10.1038/nature01905) 12955148

[198] Wang J-Q et al. 2004 TDAG8 is a proton-sensing and psychosine-sensitive G-protein-coupled receptor. J. Biol. Chem. 279, 45 626–45 633. (10.1074/jbc.M406966200) 15326175

[199] Murakami N, Yokomizo T, Okuno T, Shimizu T 2004 G2A is a proton-sensing G-protein-coupled receptor antagonized by lysophosphatidylcholine. J. Biol. Chem. 279, 42 484–42 491. (10.1074/jbc.M406561200) 15280385

[200] Mashiko M, Kurosawa A, Tani Y, Tsuji T, Takeda S In press. GPR31 and GPR151 are activated under acidic conditions. J. Biochem. (10.1093/jb/mvz042) 31119277

[201] Liu J-P et al. 2010 Each one of certain histidine residues in G-protein-coupled receptor GPR4 is critical for extracellular proton-induced stimulation of multiple G-protein-signaling pathways. Pharmacol. Res. 61, 499–505. (10.1016/j.phrs.2010.02.013) 20211729

[202] Mochimaru Y, Negishi J, Murakami S, Musha S, Sato K, Okajima F, Tomura H 2018 Metals differentially activate ovarian cancer G protein-coupled receptor 1 in various species. Zool. Sci. 35, 109–114. (10.2108/zs170145) 29623784

[203] Musha S, Nagayama S, Murakami S, Kojima R, Deai M, Sato K, Okajima F, Ueharu H, Tomura H 2019 Protons differentially activate TDAG8 homologs from various species. Zool. Sci. 36, 105–111. (10.2108/zs180128) 31120644

[204] Huang C-W, Tzeng J-N, Chen Y-J, Tsai W-F, Chen C-C, Sun W-H 2007 Nociceptors of dorsal root ganglion express proton-sensing G-protein-coupled receptors. Mol. Cell. Neurosci. 36, 195–210. (10.1016/j.mcn.2007.06.010) 17720533

[205] Yang LV, Radu CG, Roy M, Lee S, McLaughlin J, Teitell MA, Iruela-Arispe ML, Witte ON 2007 Vascular abnormalities in mice deficient for the G protein-coupled receptor GPR4 that functions as a pH sensor. Mol. Cell. Biol. 27, 1334–1347. (10.1128/MCB.01909-06) 17145776 PMC1800706

[206] Le LQ, Kabarowski JH, Weng Z, Satterthwaite AB, Harvill ET, Jensen ER, Miller JF, Witte ON 2001 Mice lacking the orphan G protein-coupled receptor G2A develop a late-onset autoimmune syndrome. Immunity 14, 561–571. (10.1016/S1074-7613(01)00145-5) 11371358

[207] Wirasinha RC et al. 2018 GPR65 inhibits experimental autoimmune encephalomyelitis through CD4^+^ T cell independent mechanisms that include effects on iNKT cells. Immunol. Cell Biol. 96, 128–136. (10.1111/imcb.1031) 29363187

[208] Im DS, Heise CE, Nguyen T, O'Dowd BF, Lynch KR 2001 Identification of a molecular target of psychosine and its role in globoid cell formation. J. Cell Biol. 153, 429–434. (10.1083/jcb.153.2.429) 11309421 PMC2169470

[209] Chen Y-J, Huang C-W, Lin C-S, Chang W-H, Sun W-H 2009 Expression and function of proton-sensing G-protein-coupled receptors in inflammatory pain. Mol. Pain 5, 39 (10.1186/1744-8069-5-39) 19602228 PMC2716309

[210] Sun WH, Chen CC 2016 Roles of proton-sensing receptors in the transition from acute to chronic pain. J. Dent. Res. 95, 135–142. (10.1177/0022034515618382) 26597969

[211] Huang L-YM, Gu Y 2017 Epac and nociceptor sensitization. Mol. Pain 13, 174480691771623 (10.1177/1744806917716234) PMC5495500 28580839

[212] Sanderlin EJ et al 2017 GPR4 deficiency alleviates intestinal inflammation in a mouse model of acute experimental colitis. Biochim. Biophys. Acta Mol. Basis Dis. 1863, 569–584. (10.1016/j.bbadis.2016.12.005) 27940273 PMC5222690

[213] Wang Y et al. 2018 The proton-activated receptor GPR4 modulates intestinal inflammation. J. Crohns Colitis 12, 355–368. (10.1093/ecco-jcc/jjx147) 29136128

[214] Dong L, Li Z, Leffler NR, Asch AS, Chi J-T, Yang LV 2013 Acidosis activation of the proton-sensing GPR4 receptor stimulates vascular endothelial cell inflammatory responses revealed by transcriptome analysis. PLoS ONE 8, e61991 (10.1371/journal.pone.0061991) 23613998 PMC3628782

[215] Miltz W et al. 2017 Design and synthesis of potent and orally active GPR4 antagonists with modulatory effects on nociception, inflammation, and angiogenesis. Bioorg. Med. Chem. 25, 4512–4525. (10.1016/j.bmc.2017.06.050) 28689977

[216] Onozawa Y, Fujita Y, Kuwabara H, Nagasaki M, Komai T, Oda T 2012 Activation of T cell death-associated gene 8 regulates the cytokine production of T cells and macrophages *in vitro*. Eur. J. Pharmacol. 683, 325–331. (10.1016/j.ejphar.2012.03.007) 22445881

[217] Hardin M et al. 2014 The clinical and genetic features of COPD-asthma overlap syndrome. Eur. Respir. J. 44, 341–350. (10.1183/09031936.00216013) 24876173 PMC4154588

[218] Yuan Y et al. 2019 Genetic polymorphisms of G protein-coupled receptor 65 gene are associated with ankylosing spondylitis in a Chinese Han population: a case-control study. Hum. Immunol. 80, 146–150. (10.1016/j.humimm.2018.12.001) 30529363

[219] Lassen KG et al. 2016 Genetic coding variant in GPR65 alters lysosomal pH and links lysosomal dysfunction with colitis risk. Immunity 44, 1392–1405. (10.1016/j.immuni.2016.05.007) 27287411 PMC4936415

[220] Xiao Y, Wang X-Q, Yu Y, Guo Y, Xu X, Gong L, Zhou T, Li X-Q, Xu C-D 2016 Comprehensive mutation screening for 10 genes in Chinese patients suffering very early onset inflammatory bowel disease. World J. Gastroenterol. 22, 5578–5588. (10.3748/wjg.v22.i24.5578) 27350736 PMC4917618

[221] Hang L-H et al. 2012 Activation of spinal TDAG8 and its downstream PKA signaling pathway contribute to bone cancer pain in rats. Eur. J. Neurosci. 36, 2107–2117. (10.1111/j.1460-9568.2012.08087.x) 22515300

[222] Dai S-P, Huang Y-H, Chang C-J, Huang Y-F, Hsieh W-S, Tabata Y, Ishii S, Sun W-H, 2017 TDAG8 involved in initiating inflammatory hyperalgesia and establishing hyperalgesic priming in mice. Sci. Rep. 7, 41415 (10.1038/srep41415) 28145512 PMC5286436

[223] Lin S-H, Steinhoff M, Ikoma A, Chang Y-C, Cheng Y-R, Chandra Kopparaju R, Ishii S, Sun W-H, Chen C-C 2017 Involvement of TRPV1 and TDAG8 in pruriception associated with noxious acidosis. J. Invest. Dermatol. 137, 170–178. (10.1016/j.jid.2016.07.037) 27566399

[224] Jin Y, Sato K, Tobo A, Mogi C, Tobo M, Murata N, Ishii S, Im D-S, Okajima F 2014 Inhibition of interleukin-1β production by extracellular acidification through the TDAG8/cAMP pathway in mouse microglia. J. Neurochem. 129, 683–695. (10.1111/jnc.12661) 24447140

[225] Mogi C et al. 2009 Involvement of proton-sensing TDAG8 in extracellular acidification-induced inhibition of proinflammatory cytokine production in peritoneal macrophages. J. Immunol. 182, 3243–3251. (10.4049/jimmunol.0803466) 19234222

[226] Tcymbarevich I et al. 2019 Lack of the pH-sensing receptor TDAG8 [GPR65] in macrophages plays a detrimental role in murine models of inflammatory bowel disease. J. Crohns Colitis 13, 245–258. (10.1093/ecco-jcc/jjy152) 30535144

[227] Xu Y, Casey G 1996 Identification of human OGR1, a novel G protein-coupled receptor that maps to chromosome 14. Genomics 35, 397–402. (10.1006/geno.1996.0377) 8661159

[228] Saxena H et al. 2012 The GPCR OGR1 (GPR68) mediates diverse signalling and contraction of airway smooth muscle in response to small reductions in extracellular pH. Br. J. Pharmacol. 166, 981–990. (10.1111/j.1476-5381.2011.01807.x) 22145625 PMC3417423

[229] Pera T et al. 2018 Biased signaling of the proton-sensing receptor OGR1 by benzodiazepines. FASEB J. 32, 862–874. (10.1096/fj.201700555R) 29042451 PMC5888400

[230] Xu J et al. 2018 GPR68 senses flow and is essential for vascular physiology. Cell 173, 762–775.e16. (10.1016/j.cell.2018.03.076) 29677517 PMC5951615

[231] Chandra V, Karamitri A, Richards P, Cormier F, Ramond C, Jockers R, Armanet M, Albagli-Curiel O, Scharfmann R 2016 Extracellular acidification stimulates GPR68 mediated IL-8 production in human pancreatic β cells. Sci. Rep. 11, 25765 (10.1038/srep25765) PMC4863151 27166427

[232] Tomura H et al. 2005 Prostaglandin I_2_ production and cAMP accumulation in response to acidic extracellular pH through OGR1 in human aortic smooth muscle cells. J. Biol. Chem. 280, 34 458–34 464. (10.1074/jbc.M505287200) 16087674

[233] Tomura H et al. 2008 Cyclooxygenase-2 expression and prostaglandin E_2_ production in response to acidic pH through OGR1 in a human osteoblastic cell line. J. Bone Miner. Res. 23, 1129–1139. (10.1359/jbmr.080236) 18302504

[234] Ichimonji I et al. 2010 Extracellular acidification stimulates IL-6 production and Ca^2+^ mobilization through proton-sensing OGR1 receptors in human airway smooth muscle cells. Am. J. Physiol. Lung Cell. Mol. Physiol. 299, L567–L577. (10.1152/ajplung.00415.2009) 20656891

[235] Ru F, Banovcin P, Kollarik M 2015 Acid sensitivity of the spinal dorsal root ganglia C-fiber nociceptors innervating the guinea pig esophagus. Neurogastroenterol. Motil. 27, 865–874. (10.1111/nmo.12561) 25846134 PMC4446164

[236] de Vallière C et al. 2016 Hypoxia positively regulates the expression of pH-sensing G-protein-coupled receptor OGR1 (GPR68). Cell. Mol. Gastroenterol. Hepatol. 2, 796–810. (10.1016/j.jcmgh.2016.06.003) 28174749 PMC5247318

[237] de Vallière C et al. 2015 G protein-coupled pH-sensing receptor OGR1 is a regulator of intestinal inflammation. Inflamm. Bowel Dis. 21, 1269–1281. (10.1097/MIB.0000000000000375) 25856770 PMC4450952

[238] Radu CG, Nijagal A, McLaughlin J, Wang L, Witte ON 2005 Differential proton sensitivity of related G protein-coupled receptors T cell death-associated gene 8 and G2A expressed in immune cells. Proc. Natl Acad. Sci. USA 102, 1632–1637. (10.1073/pnas.0409415102) 15665078 PMC545089

[239] Huang Y-H, Su Y-S, Chang C-J, Sun W-H 2016 Heteromerization of G2A and OGR1 enhances proton sensitivity and proton-induced calcium signals. J. Recept. Signal Transduct. Res. 36, 633–644. (10.3109/10799893.2016.1155064) 27049592

[240] Hohmann SW, Angioni C, Tunaru S, Lee S, Woolf CJ, Offermanns S, Geisslinger G, Scholich K, Sisignano M 2017 The G2A receptor (GPR132) contributes to oxaliplatin-induced mechanical pain hypersensitivity. Sci. Rep. 7, 446 (10.1038/s41598-017-00591-0) 28348394 PMC5428564

[241] Su Y-S, Huang Y-F, Wong J, Lee C-W, Hsieh W-S, Sun W-H 2018 G2A as a threshold regulator of inflammatory hyperalgesia modulates chronic hyperalgesia. J. Mol. Neurosci. 64, 39–50. (10.1007/s12031-017-1000-3) 29159784

[242] Reinhold AK, Batti L, Bilbao D, Buness A, Rittner HL, Heppenstall PA 2015 Differential transcriptional profiling of damaged and intact adjacent dorsal root ganglia neurons in neuropathic pain. PLoS ONE 10, e0123342 (10.1371/journal.pone.0123342) 25880204 PMC4400143

[243] Coddou C, Stojilkovic SS, Huidobro-Toro JP 2011 Allosteric modulation of ATP-gated P2X receptor channels. Rev. Neurosci. 22, 335–354. (10.1515/rns.2011.014) 21639805 PMC3647606

[244] Peters CH, Ghovanloo M-R, Gershome C, Ruben PC 2018 pH modulation of voltage-gated sodium channels. In Voltage-gated sodium channels: structure, function and channelopathies (Internet) (ed. Chahine M), pp. 147–160. Cham, Switzerland: Springer International Publishing See http://link.springer.com/10.1007/164_2018_99. 10.1007/164_2018_99 29460150

[245] Liu Z, Wang W, Zhang T-Z, Li G-H, He K, Huang J-F, Jiang X-L, Murphy RW, Shi P 2013 Repeated functional convergent effects of Na_V_1.7 on acid insensitivity in hibernating mammals. Proc. R. Soc. B 281, 20132950 (10.1098/rspb.2013.2950) PMC3871328 24352952

[246] Hockley JRF, Taylor TS, Callejo G, Husson ZM, Smith EStJ 2019 Acid and inflammatory sensitisation of naked mole-rat colonic afferent nerves. *bioRxiv* (Internet). 17 May 2019 (cited 20 May 2019); See http://biorxiv.org/lookup/doi/10.1101/636571.

[247] Hockley JRF et al. 2017 Visceral and somatic pain modalities reveal Na_V_1.7-independent visceral nociceptive pathways. J. Physiol. 595, 2661–2679. (10.1113/JP272837) 28105664 PMC5390874

